# Motor cortical areas facilitate schema-mediated integration of new motor information into memory

**DOI:** 10.1162/IMAG.a.1203

**Published:** 2026-04-13

**Authors:** Serena Reverberi, Nina Dolfen, Bradley Ross King, Geneviève Albouy

**Affiliations:** Department of Movement Sciences, Movement Control and Neuroplasticity Research Group, KU Leuven, Leuven, Belgium; Defitech Chair of Clinical Neuroengineering, INX and BMI, EPFL Valais, Clinique Romande de Réadaptation, Sion, Switzerland; Faculty of Psychology and Educational Sciences, Department of Experimental Psychology, Ghent University, Ghent, Belgium; Department of Health and Kinesiology, College of Health, University of Utah, Salt Lake City, UT, United States

**Keywords:** motor learning, schema, integration, fMRI, memory consolidation

## Abstract

New information is rapidly learned when it is compatible with pre-existing knowledge, that is, with a previously acquired schematic representation of the learned information. The influence of pre-established schema on learning has been extensively studied in the declarative memory domain, where it was shown that schema-compatible information could be rapidly assimilated into neocortical storage, bypassing the slow hippocampo-neocortical memory transfer process. Schema-mediated learning was recently examined in the motor memory domain; however, its neural substrates remain unknown. The goal of this study was to address this knowledge gap using both univariate and multivariate analyses of functional magnetic resonance imaging (fMRI) data acquired in 60 young healthy participants during the practice of a motor sequence that was either compatible or incompatible with a previously acquired cognitive–motor schema. Consistent with the literature, our behavioural results suggest that performance of sequential movements was enhanced when practice occurred in a context that was compatible with the previously acquired schema. Brain imaging results show that practice in a schema-compatible context specifically recruited the left primary motor cortex and resulted in a decrease in connectivity between the bilateral motor cortex and a set of task-relevant brain regions including the hippocampus, striatum, and cerebellum. Temporally fine-grained MRI analyses suggest that multivoxel activation patterns in the primary motor and the premotor cortices were modulated by schema-compatibility, with greater pattern similarity detected for sequence elements corresponding to and surrounding novel sequential movements under schema-compatible compared with schema-incompatible conditions. Altogether, these results suggest that motor cortical regions facilitate schema-mediated integration of novel movements into memory.

## 1 Introduction

The overlap of multiple memory traces related in content is believed to result in the development of a cognitive schema, defined as an associative knowledge structure consisting of the gist representation of previous experiences (Lewis & Durrant, 2011). The availability of an established schema has been demonstrated to facilitate the learning of new information that is compatible with this previous knowledge (van Kesteren et al., 2012). Experimental evidence supporting the schema model of memory consolidation initially came from rodent work (Tse et al., 2007, 2011). After learning a specific pattern of flavour–location associations in an arena, rats were subsequently able to rapidly consolidate novel associations if they were compatible (but not if they were incompatible) with the previously learned set of flavour–location associations (Tse et al., 2007). Schema-facilitated learning was subsequently demonstrated in humans in the declarative memory domain. Memory performance was consistently shown to be enhanced when learning occurred in schema-compatible—compared to schema-incompatible—conditions for a wide range of stimuli including object–scene associations (van Kesteren et al., 2013), face–location, and face–house associations (Atienza et al., 2011; Liu et al., 2018), item–color associations (Cycowicz et al., 2008), noun–adjective associations (Bein et al., 2015), auditory–visual associations (Heikkilä et al., 2015), study-related facts (van Kesteren et al., 2014), and movie clips (Keidel et al., 2018; van Kesteren et al., 2010).

Several neurobiological models of schema-mediated learning have been proposed in the declarative memory domain (Lewis & Durrant, 2011; Sekeres et al., 2024; van Kesteren et al., 2012). These models attempt to reconcile the rapid memory consolidation observed under schema-compatible conditions with the standard systems consolidation model suggesting a gradual and slow consolidation process which can span days to years (Squire & Alvarez, 1995). According to this standard model of consolidation, the hippocampus is necessary for the initial acquisition of novel memories, which are then slowly transferred to long-term neocortical storage sites, becoming independent of hippocampal activation (Squire & Alvarez, 1995). Schema models of consolidation propose that this slow transfer process can be bypassed when the novel information to be learned is compatible with previously acquired knowledge (Lewis & Durrant, 2011; Sekeres et al., 2024; van Kesteren et al., 2012). Consistent with this view, patients with hippocampal damage were able to learn associative information by leveraging existing semantic structures (Sharon et al., 2011). Furthermore, neuroimaging studies have demonstrated that the encoding of schema-compatible memories results in increased activity in the medial prefrontal cortex (mPFC) while learning schema-incompatible information recruits the hippocampus (Bonasia et al., 2018; van Kesteren et al., 2013). In the same vein, both the mPFC (Audrain & McAndrews, 2022) and the angular gyrus (Wagner et al., 2015) have been shown to play critical roles in the retrieval of schema-compatible memories and rule-based schema memories. Last, in line with the idea that the integration of novel memories compatible with pre-existing schema bypasses slow hippocampo-cortical transfer of information, connectivity studies have shown that the integration of schema-compatible, versus schema-incompatible, information resulted in reduced functional connectivity between the hippocampus and the mPFC (van Kesteren et al., 2010, 2014).

The schema model of memory consolidation was only more recently examined in the non-declarative memory domain. For example, a study on perceptual memory showed that the learning of new melodies was enhanced for participants of Western culture when the melodies conformed to a tone distribution pattern commonly found in Western music (Durrant et al., 2015). In the motor memory domain, it has been proposed that schematic representations of motor sequences can develop after learning, and that the corresponding cognitive–motor schema encompasses the association between the performed movements and their ordinal position within the sequence stream (King et al., 2019). In line with observations from other memory domains, previous work demonstrated that a novel sequence whose ordinal structure was compatible with the previously acquired motor sequence schema was learned significantly faster than a schema-incompatible sequence (King et al., 2019). Overall, evidence from the behavioural studies reviewed above suggests that the schema model of memory presents similar characteristics across memory domains. However, it remains unknown whether this similarity extends to the underlying neural processes, as the cerebral substrates subtending schema-mediated learning have only been investigated in the declarative memory domain. Traditional models of motor learning propose that initial rapid learning is supported by regions such as the hippocampus, associative striatum, and cerebellum as well as their cortical projections (Albouy et al., 2013; Dayan & Cohen, 2011; Doyon et al., 2003, 2009; Penhune & Steele, 2012). This rapid initial acquisition is then thought to be followed by a slow learning phase, with long-term retention of motor sequences eventually supported by the motor, premotor, and parietal cortices (Berlot et al., 2020; Doyon et al., 2009; Yokoi & Diedrichsen, 2019). Network-wise, motor learning was shown to be accompanied by an initial enhancement in inter- and intrahemispheric connectivity (Sun et al., 2007), followed by a global reduction in network interactions (Tzvi et al., 2014, 2015). The neural correlates of accelerated, schema-mediated motor learning remain, however, to be determined.

The goal of the current investigation was, therefore, to examine the neural processes supporting schema-mediated learning in the motor memory domain. To do so, we used both univariate and multivariate analyses of functional magnetic resonance imaging (fMRI) data acquired during the learning of novel motor sequences that were either compatible or incompatible with a pre-existing cognitive–motor schema. Our overarching hypothesis was that the learning of schema-incompatible motor sequences would recruit brain areas traditionally involved in the *learning of new motor sequences* and would, therefore, rely on increased activity and connectivity in the cerebellum, striatum, and the hippocampus (as well as their cortical projections). Additionally, we expected neural patterns in the regions listed above and involved in novel learning to show increased similarity for novel sequential elements when these are learned under schema-incompatible, but not schema-compatible, conditions. In contrast, we expected that learning schema-compatible sequences would recruit regions involved in the *cortical storage of previously learned motor sequences* such as the primary motor cortex (M1) as well as the premotor and parietal cortices, and would be accompanied by decreased connectivity between these storage cortical regions and the brain areas involved in novel motor learning described above. Finally, we expected multivariate patterns in these storage regions to be more similar for novel sequential elements learned under schema-compatible, as compared with schema-incompatible, conditions.

## 2 Methods

### 2.1 Participants

Sixty healthy young volunteers (mean age: 23 years; age range: 19–30 years old) were recruited from KU Leuven and surroundings. The following criteria were used to determine participant inclusion: (1) right handed, as assessed with the Edinburgh Handedness Inventory (Oldfield, 1971); (2) no prior extensive training with a musical instrument requiring dexterous finger movements (e.g., piano, guitar) or as a professional typist; (3) free of medical, neurological, psychological, or psychiatric conditions, including depression and anxiety as assessed by the Beck’s Depression and Anxiety Inventories (Beck et al., 1988, 1996); (4) no indications of abnormal sleep, as assessed by the Pittsburgh Sleep Quality Index (PSQI (Buysse et al., 1989)); (5) not considered extreme morning or evening types, as quantified with the Horne & Ostberg chronotype questionnaire (Horne & Ostberg, 1976); (6) free of psychoactive and sleep-influencing medications; (7) non-habitual smokers; and (8) not having completed trans-meridian trips or worked night shifts in the month prior to participation. Participants were additionally excluded from participation in the study if they reported intake of prescription medication or natural/over-the-counter products with psychoactive or sleep-disturbing effects, or if they reported having current or past drug consumption issues. Finally, they were asked to confirm, before their enrolment in the study, their ability to refrain from intake of drugs, alcohol, and cigarettes during the experimental days. Written informed consent was obtained from all participants for being included in the study, and monetary compensation was given for their participation. Participants were pseudo-randomly assigned to the schema-compatible (COMP) or schema-incompatible (INCOMP) group, with an equal gender ratio between groups (each group included 20 female and 10 male participants; see Supplementary Table S1 for details on participants demographics).

#### 2.1.1 Sample size justification

Our sample size was based on a main effect of group (COMP vs. INCOMP) measured on the response time for novel transitions in our previous behavioural experiment employing a similar experimental design (King et al., 2019). This group main effect (F(1,36) = 5.29, *p* = 0.027, ηp^2^ = 0.128, Cohen’s F = 0.383) resulted in an estimated minimum inclusion of 21 subjects per group, as assessed via G*Power (Faul et al., 2007) (Effect size f = 0.383, tails = 2; alpha = 0.05, power = 0.80, correlation among repeated measures = 0.72). As we expected performance measured in the MRI scanner to be more variable than when measured outside the scanner in a behavioural study, we aimed for an increased sample size of 30 subjects per group (60 participants in total).

### 2.2 Motor task

Participants performed an explicit serial reaction time task (SRTT; Nissen & Bullemer, 1987) coded and implemented in MATLAB (Mathworks Inc., Sherbom, MA) using Psychophysics Toolbox version 3 (Kleiner et al., 2007). Participants performed the task on a computer keyboard while either seated in front of a laptop screen (Session 1, see experimental procedure) or lying on their back in the MRI scanner, on a specialized MR-compatible keyboard (Session 2). In both sessions, the participants were not able to see their fingers.

**Fig. 1.**
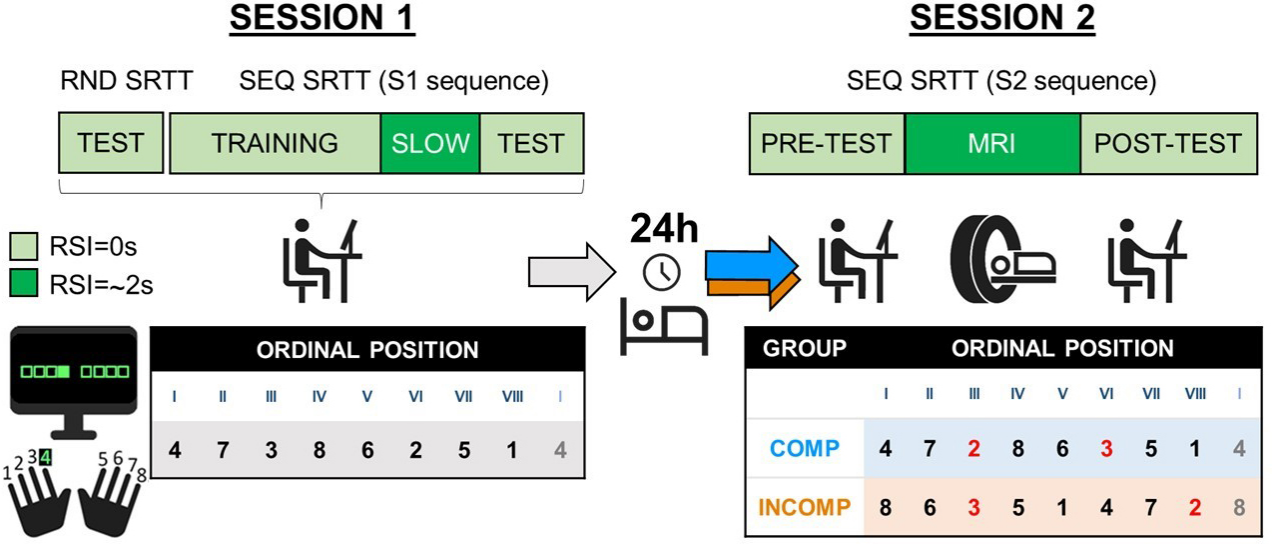
Task and procedure. Participants completed two experimental sessions separated by 24 h, in which they performed different SRT tasks. In Session 1 (S1), participants were seated at a desk and completed a pseudo-random SRTT (RND) test to assess baseline performance, followed by the sequential SRTT (SEQ; divided into training, slow, and test sections), during which the cognitive–motor schema of S1 sequence was acquired. Participants were instructed to press the key shown on-screen with the corresponding finger as fast and as accurately as possible. Participants learned the sequence 4-7-3-8-6-2-5-1 (1 to 8: left to right little finger) shown in the table, with the roman numerals (I to VIII) representing the ordinal position of the keys/fingers to be pressed in the sequence. In Session 2, participants learned a new motor sequence (referred to as S2 sequence). They performed this sequential SRTT during a pre-test, followed by the MRI session, and then a post-test (pre- and post-tests were performed outside the MRI scanner). The S2 sequence was created by switching two keys of the S1 sequence (referred to as “novel” keys, shown in red in Session 2 table (right panel)). The compatibility to the previously learned ordinal structure was manipulated by changing the starting point of the S2 sequence between the different groups such that the sequence was performed in an either highly compatible or incompatible ordinal framework in the COMP and INCOMP groups, respectively (see Section 2.3 for details). SRT tasks were performed with a response to stimulus interval (RSI) of 0 s (light green), or of 2 s on average (dark green); the latter being used to optimize the MRI analyses (see Section 2.5.2.3 for details). Icons used in this figure were adapted from Google Material Symbols (Apache License, version 2.0).

During the task, participants were presented with eight squares on the computer screen, corresponding spatially to the eight fingers used to perform the task (thumbs were not used, see Fig. 1). The outline of the squares was coloured red or green during periods of rest or practice, respectively. During practice, cues (full green squares, see Fig. 1) consecutively appeared in the different spatial locations on the screen, and participants were instructed to press the corresponding key as fast and as accurately as possible. The order of these visual cues followed either a pseudorandom pattern (random SRTT) or a deterministic eight-element sequence pattern (sequential SRTT). In the latter case, participants were informed that the order of key presses would follow a sequential pattern but were not given any additional information about the sequence (e.g., sequence length, number of repetitions). For the pseudorandom condition, each of the eight keys/fingers was used once every eight keypresses, ensuring that the distribution of keys/fingers was the same as in the sequence condition.

The number of key presses per practice block (64 vs. 96), the duration of the rest blocks (15 s vs. 10 s), and the response–stimulus interval (RSI = 0 s vs. 2 s [jittered between 1.5–2.5 s]) were different when the task was performed inside or outside the scanner (see Section 2.3) in order to optimize the analyses of the MRI data (see Sections 2.3. and 2.5.2.3. below).

### 2.3 Experimental design

The experimental design was similar to our previous work (King et al., 2019; Reverberi et al., 2023) and is shown in Figure 1. Participants completed two sessions of the serial reaction time task separated by a delay of approximately 24 h. All participants were instructed to follow a regular sleep/wake schedule according to their own preferred rhythm (i.e., ±1 h of their habitual bed and wake time with bedtime no later than 1AM and a minimum 7 h of sleep per night) for the three nights prior to Session 1 and the night between the two experimental sessions. Compliance to this schedule was assessed with sleep diaries for the three nights leading up to the experiment and with wrist actigraphy recordings (ActiGraph, Pensacola, USA) for the night between the two experimental sessions. Sleep quantity and quality for the nights preceding each experimental session were also assessed via the St. Mary’s sleep questionnaire (Ellis et al., 1981). At the start of each experimental session, participants were also asked to confirm they had not (i) taken any psychoactive prescription or over-the-counter medication in the past 3 days, (ii) consumed alcohol or undertaken strenuous exercise in the past 24 h, (iii) consumed any caffeine products the morning of the experiment, and (iv) consumed tobacco or nicotine-containing products in the 4 h preceding the experimental session. Vigilance was estimated at the start of each session with the psychomotor vigilance task (PVT, Dinges & Powell, 1985). Results related to sleep and vigilance data are reported in Supplementary Table S1.

During Session 1 (S1), which took place outside the scanner, all participants learned the same motor sequence (Sequence 1; 4-7-3-8-6-2-5-1, in which 1 and 8 represent the left and right little fingers, without the thumbs). During Session 2 (S2), in line with our previous research (King et al., 2019; Reverberi et al., 2023), all participants learned a new motor sequence that was presented in an ordinal context that was either highly compatible (COMP group) or highly incompatible (INCOMP group) with the cognitive–motor schema established during Session 1 and consisting of the ordinal structure of the previously learned Sequence 1 (King et al., 2019). Specifically, the sequence performed during Session 2 (S2) was identical to Sequence 1 with the exception that the position of two keys was switched (keys 2 and 3, “novel” keys in-text and highlighted in red in Fig. 1). The starting point of the S2 sequences was different between groups such that the sequences were performed either in a highly compatible (COMP) or incompatible (INCOMP) ordinal framework. Specifically, the ordinal structure of the S2 sequence (4-7-2-8-6-3-5-1) in the COMP group was highly compatible to that of Sequence 1 as 75% of the keys were presented in the same ordinal position as S1 and thus 75% of key/ordinal position pairings learned in Sequence 1 were preserved. In contrast, in the INCOMP group, the S2 sequence (8-6-3-5-1-4-7-2) contained only 12.5% of keys that were presented in the same ordinal position as S1 (i.e. a single key/ordinal position pairing was preserved from Sequence 1).

In each session, the sequence task was divided into multiple sections. During S1, participants first performed four blocks of a pseudo-random SRTT (*random*) to assess general motor execution. This was followed by the execution of a sequential SRTT (*training*: 20 blocks, *slow*: 2 blocks, and *test*: 4 blocks). In the training and test blocks, the task was performed with RSI = 0 s with a design that is identical to that of our previous work (King et al., 2019; Reverberi et al., 2023). In the slow blocks, RSI was set to ~2 s (jittered between 1.5 and 2.5 s) to familiarize participants with the task design used in the MRI scanner the subsequent day (see below). The test blocks were administered approximately 1 min after the slow runs to assess end-of-training performance following the dissipation of mental and physical fatigue (Pan & Rickard, 2015). During S2, participants performed three sections of the sequential SRTT. These included a *pre-test* outside the scanner (four blocks), which was followed by eight runs of task performed in the MRI scanner (*MRI*), and finally a *post-test* including eight practice blocks outside the scanner. Pre- and post-tests were performed with RSI = 0 s to assess performance outside the scanner with a similar design as during S1, that is, without the timing constraints of the MRI session. The task in the scanner was performed with a slower RSI of ~2 s (jittered between 1.5 and 2.5 s) to optimize event-related MRI analyses.

For the sections of task referred to as *random, training, test, pre-test,* and *post-test* (see Fig. 1), performed outside of the MRI scanner in Sessions 1 and 2, each block of practice contained 64 key presses (corresponding to 8 repetitions of the 8-element sequence or 8 pseudo-random key presses) and was separated from the next block by a rest period of 15 s. The sections of task referred to as *slow* in Session 1 and *MRI* in Session 2 contained 2 and 8 practice runs, respectively, with each run including 96 keypresses (corresponding to 12 repetitions of the sequence) and three 10-s rest periods randomly inserted between sequence repetitions to minimize fatigue.

### 2.4 fMRI acquisition and preprocessing

FMR images were acquired using a Phillips Achieva 3.0T MRI System equipped with a 32-channel head coil. Structural T1-weighted images were acquired with a 3D MP-RAGE sequence (TR = 9.5 ms, TE = 4.6 ms, TI = 858.1 ms, FA = 9°, 160 slices, FoV = 250 × 250 mm^2^, matrix size = 256 × 256 × 160, voxel size = 0.98 × 0.98 × 1.20 mm^3^). Functional images were acquired during the eight task runs with a T2* gradient echo-planar sequence using axial slice orientation that covers the whole brain (TR = 2000 ms, TE = 30 ms, FA = 90°, 54 transverse slices, 2.5 mm slice thickness, 0.2 mm inter-slice gap, FoV = 210 × 210 mm^2^, matrix size = 84 × 82 × 54 slices, voxel size = 2.5 × 2.56 × 2.5 mm^3^).

Functional data were preprocessed using statistical parametric mapping (SPM12; Welcome Department of Imaging Neuroscience, London, UK) implemented in MATLAB. Functional images were first slice-time corrected using the middle slice as reference slice. Functional time series were then realigned with a two-step approach using rigid body transformations iteratively optimized to minimize the residual sum of squares between each functional image and the first image of its corresponding run in a first step, and between each functional image and the across-run mean functional image in a second step. Analysis of the realignment parameters across all included runs indicates minimal head movement during scanning, as the mean maximum translation in the three directions in our sample was of 0.46 ± 0.28 mm in the COMP group and of 0.41 ± 0.30 mm in the INCOMP group (no difference between groups, independent t-test *t* = 0.54, *p* = 0.59). Note that imaging runs were excluded from data analyses if the maximum movement within the run exceeded ~2 voxels (i.e., 5 mm) in any of the three dimensions (see Section 2.5.2.1 for details about excluded runs). Preprocessing further included co-registration of the pre-processed functional images to the structural T1-image using rigid body transformation optimized to maximize the normalized mutual information between the functional and anatomical images. The T1 image was then segmented into grey matter, white matter, cerebrospinal fluid, bone, soft tissue, and background, and each participant’s forward deformation field was used for the normalization step (univariate analyses only). For the univariate analyses, structural and functional data were then normalized to MNI space (resampling size of 2 x 2 x 2mm) and functional data were spatially smoothed (isotropic Gaussian kernel, 8 mm full-width half-maximum). For the multivariate analyses, data were maintained in native space to account for inter-individual variability in the topography of memory representations (Haxby et al., 2001).

### 2.5 Statistical analyses

#### 2.5.1 Behavioural analyses

All statistical analyses on behavioural data were performed using IBM SPSS Statistics for Windows, version 28 (IBM Corp, 2021). In case of violation of the sphericity assumption, we applied Greenhouse–Geisser corrections for ε ≤ 0.75, Huynh–Feldt corrections for ε > 0.75 (Verma, 2015).

##### 2.5.1.1 Behavioural data exclusions

Due to experimental computer malfunction resulting in missing task blocks, (i) one participant of the INCOMP group was excluded from the analyses of Session 1 training data (Session 1 training analyses, therefore, included 59 participants, 30 in the COMP and 29 in the INCOMP group); (ii) another participant of the INCOMP group was excluded from the analyses of Session 1 test data (which, therefore, included 59 participants, 30 in the COMP and 29 in the INCOMP group); and (iii) 1 participant of the COMP group was excluded from the analyses of Session 2 pre-test data (which, therefore, included 59 participants, i.e., 29 in the COMP and 30 in the INCOMP group). One participant in the INCOMP group was excluded from the analyses of the behavioural data collected in the MRI scanner as they did not follow task instructions during one run of the task (large lags between responses due to drowsiness; Session 2 MRI behavioural analyses, therefore, included 59 participants, 30 in the COMP and 29 in the INCOMP group). Note that this run was also excluded from the MRI analyses (see Section 2.5.2.1).

##### 2.5.1.2 Primary outcome variables

The primary outcome variables for the SRTT analyses were performance speed measured as the response time (RT), that is, the time between cue presentation and participant key press, and performance accuracy, that is, the percentage of correct key presses. The averaged response times for correct keypresses and the accuracy were computed for each block or run of practice on the task. Performance on the sequential SRTT was analysed with repeated measures ANOVAs separately for each section of the task (see Section 2.2 for details) using within-subject factor *block* or *run,* between-subject factor *group* (COMP/INCOMP)*,* and dependent measure *RT* or *accuracy.*

##### 2.5.1.3 Negative control analyses

Negative control analyses were performed on vigilance scores (i.e., mean reaction time on the PVT task), sleep quantity (hours) and quality (score 1–5), inclusion questionnaire scores (see below for details), and pseudo-random SRTT data (speed and accuracy measures). Group differences in sleep quantity and quality, vigilance, as well as handedness, depression, anxiety, and chronotype scores (see Section 2.1. and Supplementary Table S1 for details) were assessed with independent-samples t-tests (two-sided, equal variances assumed where Levene’s test non-significant). Group differences in the Session 1 pseudo-random SRTT, reflecting general motor execution, were assessed with a *group* x *block* ANOVA (see Supplementary Table S2).

#### 2.5.2 fMRI analyses

Univariate statistical analyses were performed with SPM12 (Welcome Department of Imaging Neuroscience, London, UK) implemented in MATLAB. Multivariate pattern analyses were performed using the CoSMoMVPA toolbox (Oosterhof et al., 2016).

##### 2.5.2.1 MRI data exclusions

One participant was excluded from all MRI data analyses due to excessive head motion in over half of the MRI runs, resulting in a final sample size of 59 participants for all MRI analyses with 30 participants for the INCOMP group and 29 for the COMP group. One single run of MR task was additionally excluded from the neuroimaging analyses for two additional participants of the INCOMP group, one due to excessive head motion and one due to non-compliance with task instructions (detailed under Section 2.5.1.1) in these particular runs. All other runs were included in the analyses.

##### 2.5.2.2 Univariate analyses

###### 2.5.2.2.1 Activation-based analyses

The univariate analysis of fMRI data collected during Session 2 was performed in two steps. In the first-level fixed effects analyses, one regressor of interest represented task practice and included one event per each key/finger cue that was modelled using stick functions (0 ms duration) locked to cue onset and convolved with the canonical haemodynamic response function. Regressors of no interest were also modelled, in particular regressors representing incorrect key presses and key presses performed during rest periods, as well as motion regressors (three translations and three rotations) derived from functional volumes realignment. Finally, the data were high-pass filtered with a cut-off period of 128 s to remove low-frequency drifts, and serial correlations in the signal were estimated using an autoregressive (order 1) plus white noise model and a restricted maximum likelihood (ReML) algorithm. Following first-level analyses, the resulting statistical parametric maps representing the contrast of task practice vs. rest were spatially smoothed for each participant (Gaussian kernel, 6 mm FWHM) and entered into second-level, random effects analyses. The second-level analyses examined brain responses during task practice versus rest across the two experimental groups using a one-sample t-test (see Supplementary Table S5) as well as the difference in neural activation between the two groups of participants (COMP and INCOMP) using a two-sample t-test (see Section 3.2 in the main text, and Supplementary Table S6 for activations outside regions of interest).

###### 2.5.2.2.2 Connectivity-based analyses

Task-related functional connectivity was examined using psychophysiological interaction (PPI) analyses. We assessed connectivity of two homologous seed regions showing a significant difference in activation between the COMP and INCOMP groups in the activation-based fMRI analysis described above (i.e., left M1, -34 -32 58 mm and right M1, 40 -20 52 mm). For each participant, singular value decomposition of the time series was used to extract the first eigenvariate of the signal across the voxels included in a 6 mm-radius sphere centred on the peaks of the activation clusters reported at the group level in each contrast of interest (COMP > INCOMP and INCOMP > COMP). General linear models (GLMs) were generated at the individual level and consisted of three regressors representing (i) the neural activity in the reference area (“physiological” regressor), (ii) the practice of the learned sequence (“psychological” regressor), and (iii) the interaction of interest between the previous two regressors. To build this interaction regressor, the underlying neuronal activity was first estimated by a parametric empirical Bayes formulation, then combined with the psychological regressor and finally convolved with the standard haemodynamic response function (Gitelman et al., 2003). Movement parameters were also included as regressors of no interest. A significant PPI reflects a change in the regression coefficients, thus a change in the strength of the functional interaction, between any reported brain region and the reference seed region, related to the practice of the learned sequence as compared with rest. Similar to the activation-based analyses described above, individual summary statistic images obtained at the first-level (fixed effects) PPI analysis were spatially smoothed (6 mm FWHM Gaussian kernel) and entered in a second-level (random-effects) analysis using a two-sample t-test to compare the strength of the functional interaction between the two groups of participants.

###### 2.5.2.2.3 Statistics

The set of voxel values resulting from each second level analysis described above (activation and functional connectivity) constituted maps of the t statistic for each contrast of interest [SPM(T)] and were thresholded for display at *p* < 0.001 (uncorrected for multiple comparisons). Statistical inferences were performed at a threshold of *p* < 0.05 after family-wise error (FWE) correction for multiple comparisons over small spherical 10-mm radius volumes centred on coordinates derived from the literature and located within regions of interest (small volume correction of the family-wise error rate (Poldrack, 2007), coordinates used for correction reported in Supplementary Table S3). Further correction for multiple volumes of interest was performed by applying the Benjamini–Hochberg correction of the false discovery rate (FDR, *p* < 0.05) (Benjamini & Hochberg, 1995) over the resulting number of significantly active brain regions listed for each contrast (see Table 3). Beta values extracted from activation- and connectivity-based analyses were graphically visualized using the MATLAB package *DataViz* (Karvelis & Oyvindlr, 2024).

##### 2.5.2.3 Multivariate analyses

The univariate analyses of the fMRI data described above allowed us to investigate group differences in brain activity/connectivity during task practice as compared with rest. We additionally performed multivariate pattern analyses to provide a more fine-grained analysis of brain responses associated with each sequential movement. This allowed us to investigate the specific effects of schema compatibility on brain responses associated with specific elements of the motor sequence.

###### 2.5.2.3.1 ROI selection

The multivariate analyses followed a procedure analogous to that of Dolfen et al. (2024) and used a region of interest (ROI) approach. The following ROIs were selected a priori, given their established involvement in motor sequence learning (Albouy et al., 2013; Berlot et al., 2020; Dayan & Cohen, 2011; Doyon et al., 2003, 2009): the hippocampus, putamen, primary motor cortex (M1), premotor cortex, and anterior superior parietal lobule (aSPL). Additionally, three exploratory ROIs were considered. The caudate nucleus, which despite its role in motor learning, did not show differential coding for ordinal, key, or key–position binding during sequence learning in previous research (Dolfen et al., 2024), and two ROIs derived from the declarative memory schema literature (i.e., the medial prefrontal cortex and the angular gyrus). The results of the corresponding exploratory analyses are presented in the Supplementary Material (Supplementary Table S9, Supplementary Fig. S1). Based on the results of the univariate analyses, the M1 ROI was divided into left and right M1, while all other ROIs were bilateral. All ROIs were anatomically defined. Subcortical ROIs (hippocampus, putamen, and caudate nucleus) were defined in native space using FSL’s automated subcortical segmentation protocol (FIRST, Patenaude et al., 2011). Cortical ROIs were created in MNI space and then converted into native space. The ROIs of M1, premotor cortex, medial prefrontal cortex, and angular gyrus were created using the Brainnettome atlas (Fan et al., 2016). M1 included the upper limb and hand function regions of Brodmann area (BA) 4. The premotor cortex (PMC) was defined as the dorsal (A6cdl; dorsal PMC) and ventral (A6cvl; ventral PMC) part of BA 6. The medial prefrontal cortex mask included the medial regions of BA 10 and BA 14 (A10m, A14m). The angular gyrus included the caudal, rostrodorsal, and rostroventral areas of BA 39 (A39c, A39rd, A39rv). The aSPL ROI was created using the Julich brain atlas (Amunts et al., 2020) and was defined to include the anterior, medial, and ventral intraparietal sulcus. An additional exclusion mask was applied to these grey matter ROIs in order to exclude any remaining voxel falling outside the brain and/or with a probability to belong to grey matter below 10%. The average number of voxels within each ROI is reported in Supplementary Table S4.

###### 2.5.2.3.2 Representational similarity analyses

Representational similarity analyses (RSA) were designed to examine the effect of schema compatibility on multivoxel activation patterns related to finger movements in their temporal position in the new sequences. For each ROI, and for each participant, representational similarity values were, therefore, calculated for each of the eight sequence elements. To do this, a GLM was created with one regressor per each key/ordinal position pairing. This resulted in a total of eight regressors of interest per run of task. For each regressor, events of interest were modelled with delta functions (0 ms duration) time locked to cue onset. Keypresses during rest and movement parameters derived from functional volume realignment were considered regressors of no interest. High-pass filtering and serial correlation estimation were performed as for the univariate analyses. The resulting statistical maps allowed the extraction of one vector of t-values (size: number of voxels per ROI) for each sequence element (i.e., each key/ordinal position pairing), each run of task, and participant. T-values were normalized by subtracting the mean t-value across all eight regressors from each voxel in each ROI, separately for each task run.

**Fig. 2.**
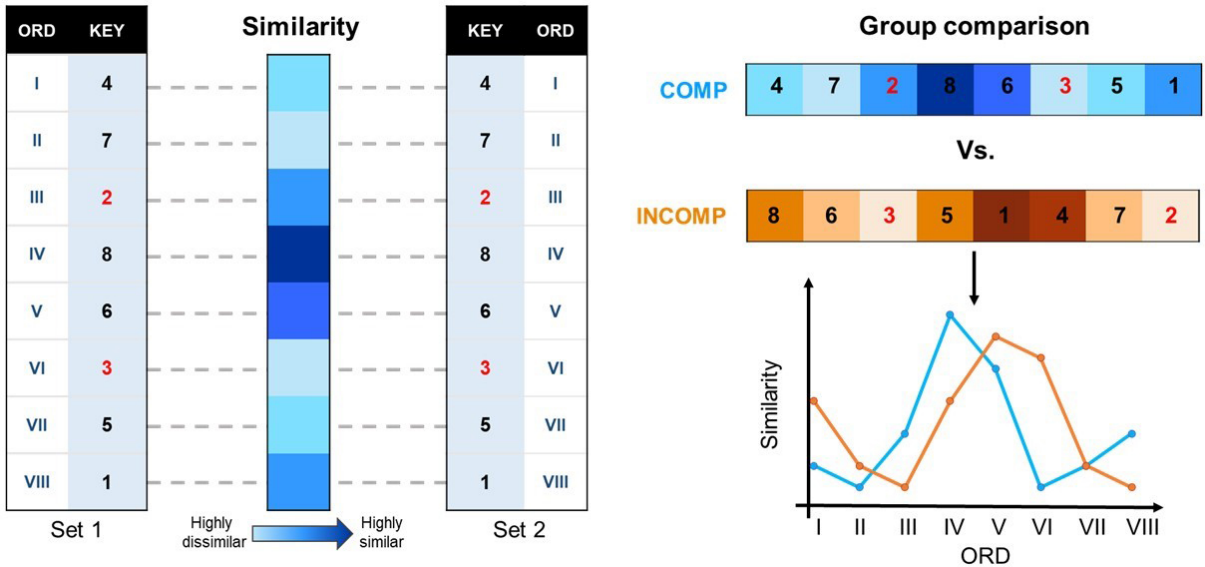
Schematic representation of the multivariate pattern analysis. Regressors modelling each sequence element were fitted to the data and used to extract vectors of t-values across voxels for each ROI, run of task, and participant (left; example for a COMP group participant). Runs were then randomly divided into two sets, and the vector similarities computed across the sets. This procedure was repeated for 70 iterations, resulting in 1 similarity value averaged across the 70 iterations per sequence element, ROI, and participant. These similarity values were then compared between experimental groups (right) for each sequence element (i.e., each key/ordinal position pairing in the sequence stream, ordered by ordinal position I through VIII) to investigate the effects of the schema compatibility manipulation on multivariate patterns in motor learning-related ROIs.

Representational similarity analyses were performed following the procedure of Dolfen et al. (2024), schematically represented in Figure 2. Specifically, Pearson correlation coefficients were computed for each regressor by correlating vectors of t-values between runs. To do so, the full dataset of eight runs was randomly divided into two independent subsets of four runs, and t-values were averaged across each subset. This resulted in two sets of eight averaged t-value vectors. Next, correlations were computed between runs for each sequence element (e.g., correlating the t-value vector corresponding to key 4/ordinal position 1 in the first set of runs and in the second set of runs). This procedure was repeated 70 times (i.e., the number of possible combinations of 8 runs divided in 2 groups of 4) and correlations were then averaged across the 70 iterations, resulting in an averaged similarity value per sequence element, ROI, and participant. Finally, the resulting correlation values were Fisher transformed. The computed similarity values represent the consistency in a brain region’s multivoxel responses to each sequential element across multiple repetitions of that element, with low and high similarity reflecting, respectively, low and high consistency of responses across repetitions and voxels in the brain region.

To investigate the effect of schema compatibility on the multivoxel activation patterns related to the sequence of movements, the computed similarity values for each ROI were entered in a 2 x 8 ANOVA with between-subject factor *group* (COMP/INCOMP) and within-subject factor *ordinal position* (I through VIII). Control analyses were performed to assess the influence of key information on the pattern of results (see Supplementary Tables S7 and S8). The Benjamini–Hochberg false discovery rate (FDR, *p* < 0.05) correction (Benjamini & Hochberg, 1995) was used to correct for testing across multiple ROIs (i.e., the six ROIs presented in the main text). When a significant interaction of group and ordinal position was detected, follow-up independent samples t-tests (two-sided, equal variances assumed where Levene’s test non-significant) were performed to examine differences in responses for each sequence element between groups. Benjamini–Hochberg FDR correction was then applied over the eight sequence elements. All statistical analyses on the representational similarity data were performed using IBM SPSS Statistics for Windows, version 28 (IBM Corp, 2021). In case of violation of the sphericity assumption, we applied Greenhouse–Geisser corrections for ε ≤ 0.75, Huynh–Feldt corrections for ε > 0.75 (Verma, 2015). Results of the RSA were graphically visualized using the MATLAB package *DataViz* (Karvelis & Oyvindlr, 2024).

## 3 Results

### 3.1 Behavioural results

**Fig. 3.**
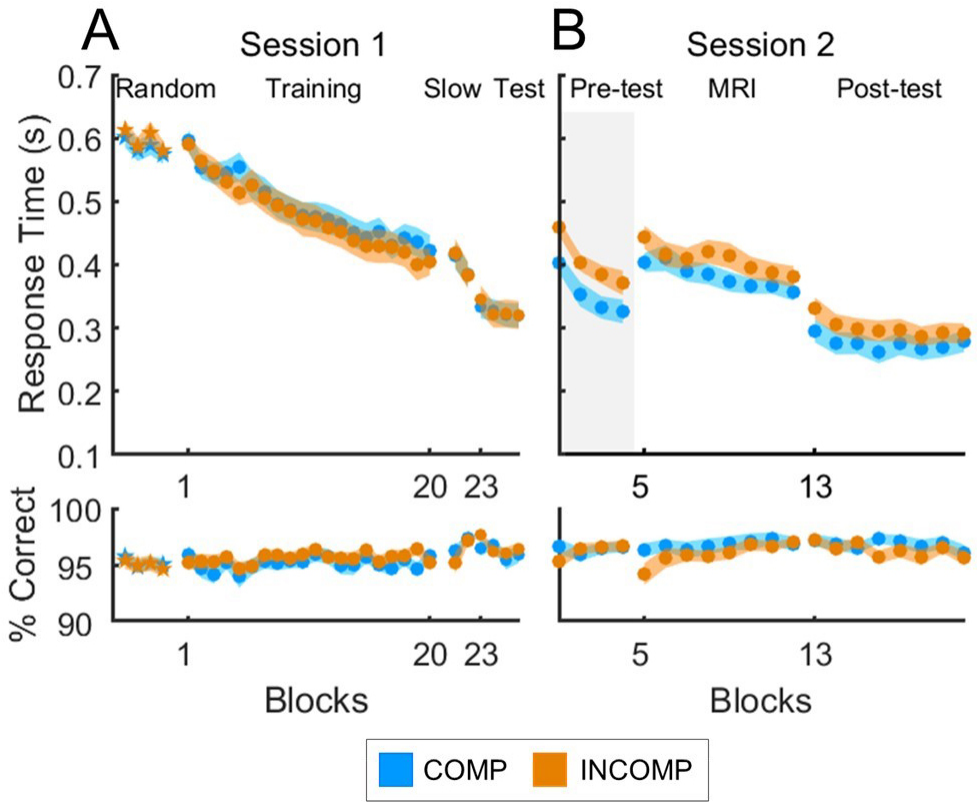
Mean response time (RT, in seconds, top panels) and accuracy (% correct keys, bottom panels) per block are shown separately for the COMP group (blue; N = 29 for S2 Pre-test, N = 30 for all other sections) and INCOMP group (orange; N = 29 for S1 Training, S1 Test, and S2 MRI, N = 30 for all other sections) during Session 1 (A) and Session 2 (B). Session 1 included both random (star markers) and sequential (circle markers) SRTT practice. Session 1 started with 4 blocks of random SRTT (response to stimulus interval, RSI = 0 s) and was followed by 20 blocks of training (RSI = 0 s), 2 blocks of slow practice (RSI = ~2 s), and 4 blocks of test (RSI = 0 s) on the sequential SRTT. Session 2 consisted of 8 blocks of practice performed in the MR scanner (RSI = ~2 s) preceded by 4 blocks of pre-test (RSI = 0 s) and followed by 8 blocks of post-test (RSI = 0 s) performed outside the scanner. Shaded coloured areas represent the standard error of the mean. A shaded grey rectangle highlights the section of the task for which a significant difference in performance was detected between groups.

The behavioural analyses focused on performance speed (response time, RT) for correct keypresses and on accuracy (% correct responses), averaged across each block or run of practice during the sequential SRTT (Fig. 3). See also Supplementary Table S2 for similar analyses of the random SRTT showing that general motor execution did not differ between groups at baseline (Fig. 3, Random).

#### 3.1.1 Session 1

Performance on the sequential SRT task during S1 is depicted in Figure 3A and the output of the corresponding statistical analyses is presented in Table 1. As expected, participants successfully learned Sequence 1, as evidenced by the significant block effect observed on performance speed across all task sections in S1. Accuracy remained overall high and stable throughout S1. Crucially, no significant group effects or block x group interactions were detected during S1 on either RT or accuracy, indicating that performance did not differ between the two experimental groups prior to the experimental manipulation introduced in S2.

**Table 1.** Sequential SRT task performance during Session 1.

	RT				Accuracy			
Effect	df	F	*p*	Part η^2^	df	F	*p*	Part η^2^
*Session 1 training*								
Block	7.63,434.72	67.91	<0.001*	0.544	12.88,734.37	1.28	0.22	0.022
Group	1,57	0.15	0.70	0.003	1,57	0.76	0.39	0.013
B x G	7.63,434.72	0.96	0.46	0.017	12.88,734.37	0.54	0.90	0.009
*Session 1 slow*								
Block	1,58	13.79	<0.001*	0.192	1,58	10.24	0.002*	0.150
Group	1,58	0.00	0.96	0.000	1,58	0.72	0.40	0.012
B x G	1,58	0.07	0.79	0.001	1,58	0.85	0.36	0.014
*Session 1 Test*								
Block	3,171	4.30	0.01*	0.070	3,171	2.98	0.03*	0.050
Group	1,57	0.01	0.94	0.000	1,57	0.45	0.51	0.008
B x G	3,171	0.81	0.49	0.014	3,171	1.29	0.28	0.022

Significant *p*-values are marked with an asterisk and bold font.

RT = response time; df = degrees of freedom; Part η^2^ = partial eta squared; BxG = block x group interaction.

#### 3.1.2 Session 2

Results suggest that participants successfully learned Sequence 2, with a significant effect of block on RT for all task sections, indicating progressively faster task performance (Fig. 3B, Table 2). In line with our hypothesized effect of schema compatibility on learning, a significant group difference (F(1,57) = 4.99, *p* = 0.03) was detected on response time during the pre-test administered prior to and outside the MR scanner, with the COMP group performing significantly faster than the INCOMP group. These results are in line with our previous research (King et al., 2019) and show an overall performance advantage when practice takes place in an ordinal framework that is compatible—as compared with incompatible—with the previously learned sequence.

**Table 2.** Sequential SRT task performance during Session 2.

	RT				Accuracy			
Effect	df	F	*p*	Part η^2^	df	F	*p*	Part η^2^
*Session 2 pre-test*								
Block	2.15,122.32	93.86	<0.001*	0.622	3,171	1.03	0.38	0.018
Group	1,57	4.99	0.03*	0.080	1,57	0.06	0.80	0.001
B x G	2.15,122.32	0.38	0.70	0.007	3,171	1.76	0.16	0.030
*Session 2 MRI*								
Block	4.39,250.12	12.18	<0.001*	0.176	4.18,238.24	3.68	0.01*	0.061
Group	1,57	1.55	0.22	0.026	1,57	1.17	0.29	0.020
B x G	4.39,250.12	1.21	0.31	0.021	4.18,238.24	1.23	0.30	0.021
*Session 2 post-test*								
Block	4.19,242.98	8.23	<0.001*	0.124	6.47,375.23	1.60	0.14	0.027
Group	1,58	1.17	0.28	0.020	1,58	0.95	0.33	0.016
B x G	4.19,242.98	0.98	0.42	0.017	6.47,375.23	1.03	0.41	0.017

Significant *p*-values are marked with an asterisk and bold font.

RT = response time; df = degrees of freedom; Part η^2^ = partial eta squared; BxG = block x group interaction.

This group difference, however, did not persist during the eight runs of practice performed in the MR scanner, when task practice was slow paced to accommodate imaging requirements, nor during the post-test performed outside of the scanner (i.e., in the same conditions as the pre-test). No group differences or block x group interactions were observed on the accuracy measure.

### 3.2 Neuroimaging results

#### 3.2.1 Univariate analyses

**Fig. 4.**
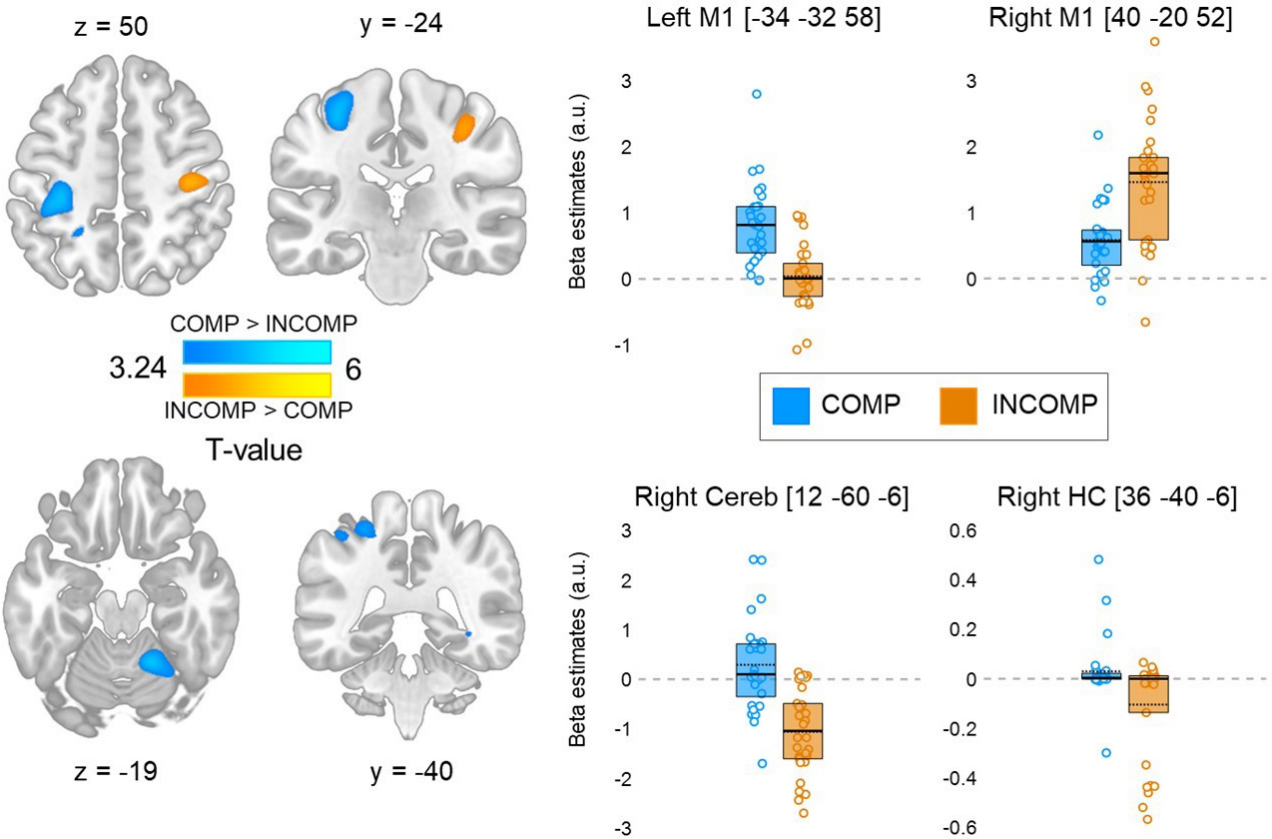
Left panel: Brain regions showing a significant difference in task-related brain activity between the COMP (N = 29) and INCOMP (N = 30) groups (blue: COMP > INCOMP and orange: INCOMP > COMP). Statistical images are thresholded at *p* < 0.001 uncorrected. Results are presented in MNI152 space. Right panel: Beta values (arbitrary units, a.u.) for the peak coordinates are displayed for the COMP (blue) and INCOMP (orange) groups. Full horizontal bars indicate the median and dotted horizontal bars the mean. Coloured circles represent individual data points. Boxes represent the interquartile range (IQR). Cereb = cerebellum; HC = hippocampus.

To assess the effect of schema compatibility on overall task-related brain activity, we compared the practice versus rest contrast between the COMP and INCOMP groups (and see Supplementary Table S5 for task-related brain activation across groups). Results show that activity in the left M1, left SMA, left precuneus, right hippocampus, and right cerebellum was significantly greater during performance of a schema-compatible, than during schema-incompatible, sequence (COMP > INCOMP, Figure 4, corresponding results presented in Table 3A). In contrast, activity in the right M1 was greater in a schema-incompatible, than in a schema-compatible, context (INCOMP > COMP, Fig. 4, Table 3B).

**Table 3.** Functional imaging results for Session 2 SRTT practice [between-group comparisons of task-related activity (A, B) and connectivity (C, D)].

Area	X	y	z	k	T	*p*_corr_
*A. Main effect of schema compatibility* (COMP > INCOMP)						
R Lingual gyrus extending into R cerebellar lobules IV-V	12	-60	-6	455	6.08	<0.003
L Primary motor cortex	-34	-32	58	406	5.23	<0.003
L Precuneus	-26	-50	54	56	3.81	0.008
L Supplementary motor area	-6	-16	60	69	4.07	0.006
R Hippocampus	36	-40	-6	10	3.43	0.017
R Cerebellar lobule VIII	18	-60	-46	14	3.36	0.017
*B. Main effect of schema incompatibility* (INCOMP > COMP)						
R Primary motor cortex	40	-20	52	253	4.37	0.001
*C. Psycho-physiological interaction, seed region left primary motor cortex* (INCOMP > COMP)						
L Intra-parietal sulcus	-50	-54	52	62	4.61	0.005
L Cerebellar lobules IV-V	-18	-50	-18	175	4.25	0.005
L Cerebellar lobules IV-V	-20	-48	-24	175	4.12	0.005
R Putamen	32	-14	0	34	4.04	0.006
L Fusiform gyrus	-40	-38	-16	62	3.79	0.008
L Hippocampus	-40	-26	-16	62	3.54	0.012
R Primary sensory cortex	30	-30	58	87	3.75	0.008
L Superior frontal cortex	-20	14	56	33	3.49	0.012
*D. Psycho-physiological interaction, seed region right primary motor cortex* (INCOMP > COMP)						
R Cerebellar lobule VI	22	-50	-24	11	3.47	0.012

Clusters outside of grey matter, clusters of size k ≤ 5, and clusters not surviving multiple comparison correction were not reported. *p*_corr_ indicates the *p*-value corrected for multiple comparisons over (i) the number of voxels in a small volume of interest (see Supplementary Table S3 for svc coordinates) using family-wise error (FWE) correction and (ii) the number of brain regions listed for each contrast using FDR correction. Significant activation outside of areas of interest is reported in the Supplementary Material (Supplementary Table S6).

**Fig. 5.**
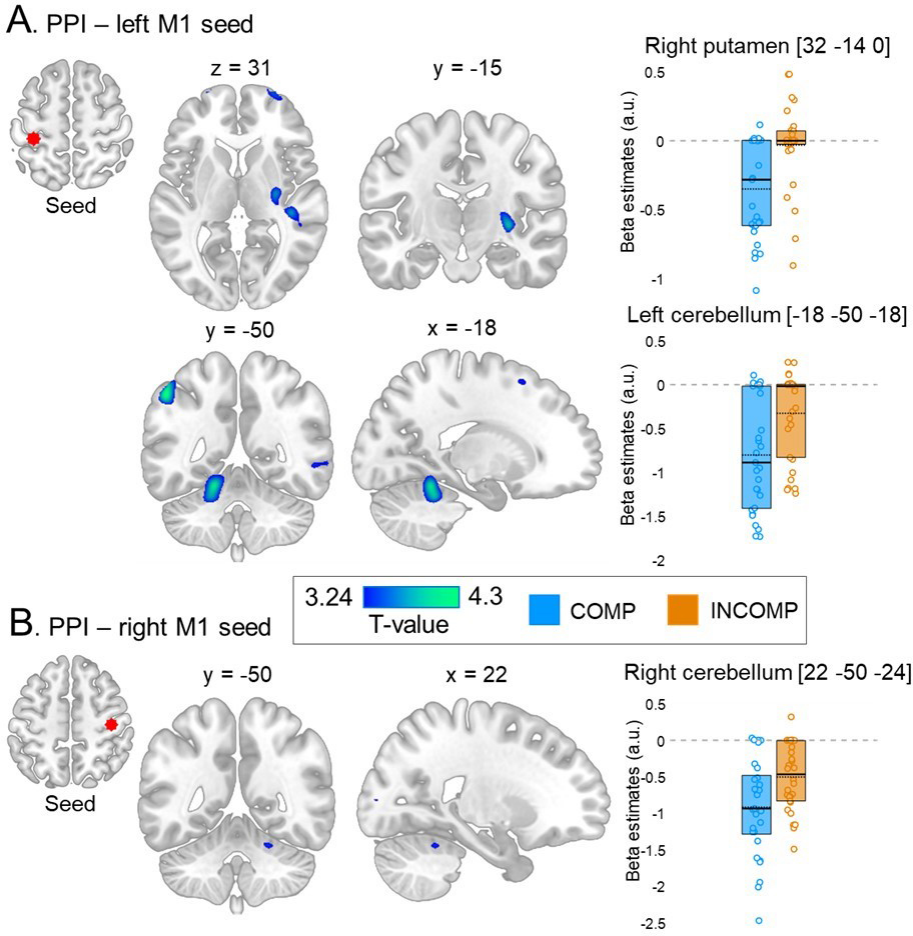
(A) Left panel: Brain regions showing a significantly lower task-related functional connectivity with the left M1 (coordinate [-34 -32 58], seed region shown in red) in the COMP (N = 29) versus INCOMP (N = 30) group. (B) Left panel: Brain regions showing a significantly lower task-related functional connectivity with the right M1 (coordinate [40 -20 52], seed region shown in red) in the COMP versus INCOMP group. In both panels, statistical images are thresholded at *p* < 0.001 uncorrected. Results are presented in MNI152 space. (A-B) Right panels: Beta values (arbitrary units, a.u.) for the peak coordinates are displayed for the COMP (blue) and INCOMP (orange) groups. Full horizontal bars indicate the median and dotted horizontal bars the mean. Coloured circles represent individual data points. Boxes represent the interquartile range (IQR).

To further investigate the lateralization observed in M1 during performance of the schema-compatible versus schema-incompatible sequence, we examined whether schema-compatibility affected the task-related functional *connectivity* of these brain regions. To do so, we performed psycho-physiological interaction analyses and tested whether the whole-brain connectivity of the M1 seeds described above (i.e., left and right M1) differed between COMP and INCOMP groups. This analysis revealed that the strength of the task-related functional connectivity between the left M1 seed and a set of regions including the left hippocampus, the right putamen, the left intra-parietal sulcus, and the left cerebellum was significantly lower in the COMP than in the INCOMP group (Fig. 5A and Table 3C). Reduced connectivity in the COMP versus INCOMP group was also observed between the right M1 seed and the right cerebellum during task practice as compared with rest (Fig. 5B and Table 3D).

In sum, the results of the univariate MRI analyses suggest that the recruitment of the right M1 is greater during sequence practice in a schema-incompatible than schema-compatible, context, while a wider set of brain regions including the left M1, hippocampus, and cerebellum is more activated in the schema-compatible, than in schema-incompatible, condition. Interestingly, both left and right M1 connectivity with striato-hippocampo-cerebellar regions was overall lower during practice in a compatible, than in a incompatible, context.

#### 3.2.2 Multivariate pattern analyses

The multivariate analyses of the fMRI data (see Section 2.5.2.3) examined group differences in brain patterns associated with each key/ordinal position pairing in the S2 sequences. Brain patterns were extracted from specific ROIs involved in motor learning, that is, the hippocampus, putamen, left and right M1, premotor cortex, and aSPL (note that all ROIs were bilateral except for M1, which was divided in left and right M1 based on the lateralization observed in the univariate analyses presented above). Exploratory analyses on three exploratory ROIs (the caudate nucleus and two ROIs) derived from the declarative memory schema literature (i.e., the medial prefrontal cortex and the angular gyrus) are presented in the Supplementary Material (Supplementary Table S9, Supplementary Fig. S1).

**Fig. 6.**
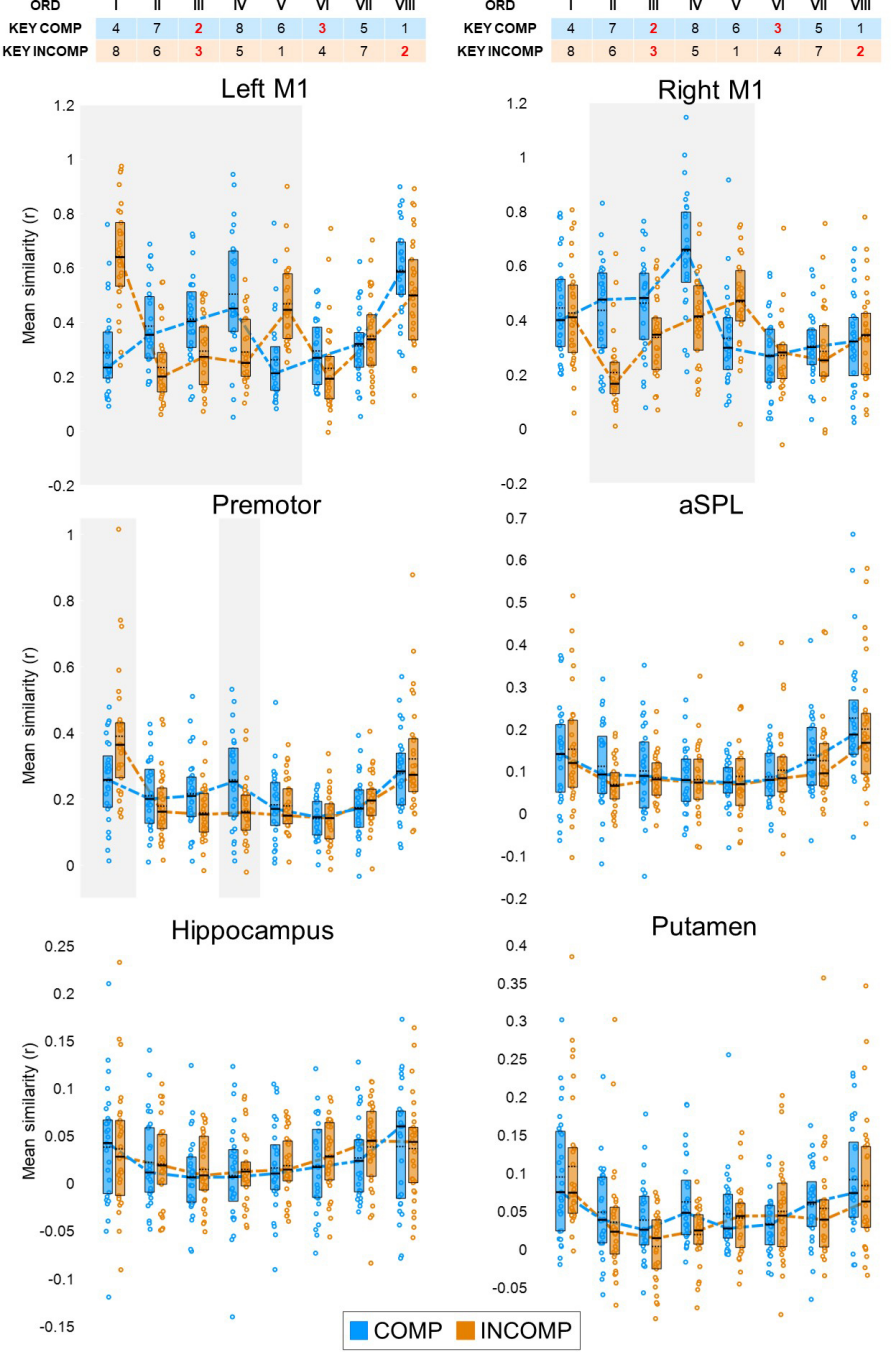
Mean pattern similarity per key/ordinal position pairing, for each experimental group and ROI. The top table represents the ordinal (ORD) position (position I to VIII) of each element in the sequence of movements (here, each number represents a finger with 1 and 8 corresponding to the left and right little fingers, respectively). Full horizontal bars indicate the median and dotted horizontal bars the mean. Coloured dashed lines connect the medians in each experimental group. Coloured circles represent individual data. Boxes represent the interquartile range (IQR). Shaded grey rectangles denote key/ordinal position pairings for which follow-up tests indicated a significant difference in similarity across groups (see Table 5). Premotor = premotor cortex; aSPL = anterior superior parietal lobule.

We opted to compare similarity patterns between groups as a function of ordinal position (rather than a function of key information) based on our previous work showing that similarity values are significantly influenced by ordinal position (Dolfen et al., 2024; but see Supplementary Material for additional analyses on the potential influence of key information on the observed pattern of results). The results of the two *groups* (COMP/INCOMP) by eight *ordinal positions* (I through VIII) ANOVAs—shown in Figure 6 and reported in Table 4—reveal a significant main effect of ordinal position in all our examined ROIs. Data inspection suggests that this effect is driven by a pronounced edge effect whereby similarity values on the first and last sequence elements tended to be greater than on all other sequence elements across most of the ROIs (i.e., the average similarity of ordinal positions I and VIII was greater than that of ordinal positions II–VII in all ROIs [paired-samples t-tests, all *p*’s < 0.01] except the right M1 [*p* = 0.35]). These results are consistent with previous work demonstrating such effect across a large number of motor sequence learning-related ROIs (Dolfen et al., 2024). In contrast, no significant main effect of group was detected in any of the examined ROIs. Interestingly, significant interactions of the factors group and ordinal position were observed in both the left and right M1 as well as in the premotor cortex, suggesting that the multivoxel activation patterns in these ROIs—but not in the aSPL, hippocampus, and putamen—were influenced by schema compatibility.

**Table 4.** Results of multivariate pattern analyses for the 2 groups (COMP/INCOMP) x 8 ordinal positions (Ord; I through VIII) ANOVAs.

	Main effect group				Main effect Ord				Group x Ord interaction			
ROI	df	F	*p*_corr_	Part η^2^	df	F	*p*_corr_	Part η^2^	df	F	*p*_corr_	Part η^2^
L M1	1,57	0.04	0.93	0.001	4.21,239.86	32.19	<0.001*	0.361	4.21,239.86	37.20	<0.002*	0.395
R M1	1,57	3.54	0.39	0.058	4.40,250.82	30.14	<0.001*	0.346	4.40,250.82	17.14	<0.002*	0.231
PMC	1,57	0.01	0.93	0.000	4.25,241.94	32.50	<0.001*	0.363	4.25,241.94	10.00	<0.002*	0.149
aSPL	1,57	0.17	0.93	0.003	3.90,222.00	18.32	<0.001*	0.243	3.90,222.00	1.00	0.49	0.017
HC	1,57	0.30	0.93	0.005	3.98,226.86	3.99	0.004*	0.065	3.98,226.86	0.28	0.89	0.005
Put	1,57	0.84	0.93	0.015	3.89,221.98	16.08	<0.001*	0.220	3.89,221.98	2.35	0.09	0.040

*p*_corr_ indicates the *p*-value corrected for multiple comparisons (FDR correction for the number of ROIs). Significant *p*-values are marked with an asterisk and bold font.

L/R M1 = left/right primary motor cortex; PMC = premotor cortex; aSPL = anterior superior parietal lobule; HC = hippocampus; Put = putamen; df = degrees of freedom; part η^2^ = partial eta squared.

To better characterize the differences in neural responses related to the sequence elements between schema-compatible and schema-incompatible conditions, we performed follow-up pairwise analyses in the three regions showing a significant group x ordinal position interaction effect (results of follow-up analyses are presented in Table 5). In both the left M1 and the premotor cortex, a significant group difference in similarity values was detected on the first sequence element such that pattern similarity was greater in the INCOMP, as compared with the COMP, group. These results suggest a greater consistency in the region’s response across repetitions of the first element of the sequence when practice takes place in a schema-incompatible, compared with a schema-compatible, context.

**Table 5.** Results of independent samples t-tests (COMP vs INCOMP) for each of the eight ordinal positions (Ord; I through VIII).

	Left M1			Right M1			Premotor cortex		
Ord	t	*p*_corr_	Cohen’s d	t	*p*_corr_	Cohen’s d	t	*p*_corr_	Cohen’s d
I	-7.74	<0.002*	-2.02	0.39	0.80	0.10	-3.22	0.01*	-0.84
II	4.08	<0.002*	1.06	5.40	<0.004*	1.41	1.16	0.42	0.30
III	3.11	0.005*	0.81	2.97	0.01*	0.77	2.24	0.08	0.58
IV	4.63	<0.002*	1.22	4.93	<0.004*	1.28	3.12	0.01*	0.82
V	-5.03	<0.002*	-1.31	-3.07	0.01*	-0.80	0.12	0.94	0.03
VI	1.71	0.11	0.45	0.03	0.98	0.01	-0.08	0.94	-0.02
VII	-0.96	0.34	-0.25	0.48	0.80	0.12	-0.97	0.45	-0.25
VIII	1.93	0.08	0.50	-0.50	0.80	-0.13	-1.13	0.42	-0.29

*p*_corr_ indicates the *p*-value corrected for multiple comparisons (FDR correction for the number of sequence elements). Significant *p*-values are marked with an asterisk and bold font.

Degrees of freedom = 57 for all tests with following exceptions: Left M1, ord IV: df = 42.41; Right M1, ord II: df = 52.20; Premotor cortex, ord IV: df = 47.60.

Additionally, multivoxel activation patterns in both left and right M1 showed increased similarity in the schema-compatible condition before, during, and after the introduction of the 1^st^ *novel* key in the sequence (2^nd^, 3^rd^, and 4^th^ sequence elements), suggesting a stable response of M1 to novel sequence elements only when these are presented in a schema-compatible context (but see Supplementary Material for the influence of key information on the results, particularly related to the 2^nd^ sequence element, and see discussion for considerations about the limitations of these control analyses).

This effect was less pronounced in the premotor cortex as it was limited to the key following the novel element (4^th^ sequence element). No significant group effects were observed on the other sequence elements including around or on the 2^nd^ novel key.

Altogether, the multivoxel activation patterns in these motor cortical regions might reflect different processes related to (i) the processing of the first sequence element under incompatible conditions and (ii) the integration of (the first) novel element into a compatible schema.

## 4 Discussion

In the present paper, we examined the neural processes underlying schema-mediated integration of novel information in motor memory. Compatibility with a previous cognitive–motor schema was examined with a protocol similar to our earlier research (King et al., 2019; Reverberi et al., 2023) in which the ordinal structure of the practiced sequence (i.e., the temporal position of movements in the motor sequence) was manipulated such that it was compatible or incompatible with that of the motor sequence learned on the previous day. Our behavioural results are in line with earlier observations (King et al., 2019) and suggest that performance on new sequential movements is enhanced in a context that is compatible with the previously acquired ordinal schema. Brain imaging results show that sequence practice in a schema-compatible (as compared with schema-incompatible) context resulted in an overall increase in left M1 activity and in a decrease in M1 connectivity with striato-hippocampo-cerebellar areas. Multivariate fMRI analyses indicated that multivoxel pattern similarity in both the left and right M1 as well as in the premotor cortex was increased around the first novel key in the sequence stream in a compatible compared with incompatible context. Altogether, these results suggest that motor cortical areas facilitate the integration of novel elements that are compatible with a pre-existing cognitive–motor schema.

Our behavioural results replicated those of our previous study (King et al., 2019), as they showed a performance advantage for the new sequence practiced in a context that was compatible, as compared with incompatible, with the ordinal framework learned the previous day. This group difference was observed during the test session prior to scanning but did not persist during the runs of task performed inside the MRI scanner. We speculate that the slower task pace in the MR scanner used to accommodate RSA might have attenuated these overall schema effect faster than in our previous research (King et al., 2019) in which both groups eventually reached the same level of performance after extensive practice. Altogether, the behavioural results confirm earlier observations of schema-mediated facilitation of the integration of new movements into motor memory (King et al., 2019).

Our activation-based univariate analyses revealed that sequence practice in a schema-compatible, versus schema-incompatible, context resulted in greater task-related activity in a set of brain regions including the left M1, the left precuneus, left SMA, right hippocampus, and right cerebellum. In contrast, practice in an incompatible context showed greater activation of the right M1. The schema-mediated lateralization effect in the primary motor cortex is intriguing as it cannot be attributed to differences in the nature of the movements performed during task practice, given that the two experimental groups performed the same bimanual sequence (only the starting points differed between groups). We suggest that these differences might instead reflect distinct cognitive processes. The literature has suggested a left hemispheric specialization in the learning of new motor skills and particularly motor sequences. Specifically, while right hemisphere recruitment has been shown to be limited to the execution of contralateral hand movements, the left hemisphere is engaged during the learning of new sequences performed with both the contralateral and the ipsilateral hands (Grafton et al., 1995, 1998, 2002). In line with these observations, left but not right hemisphere stroke has been associated with deficits in the encoding and generation of sequential movements (Harrington & Haaland, 1991; Kimura, 1977). Additionally, non-invasive brain stimulation studies showed that left M1 stimulation benefits motor learning more than sham or right M1 stimulation (Schambra et al., 2011). Thus, the heightened activity in the left M1 observed in the current study in a schema-compatible, compared with schema-incompatible, context may reflect improved sequence learning when the ordinal framework is congruent with previous knowledge. Consistent with this interpretation, task practice in a schema-compatible group also recruited a set of brain regions known to play a critical role in motor learning processes, including the SMA and the parietal cortex (Albouy et al., 2008; Doyon et al., 2009; Fernández-Seara et al., 2009; Grafton et al., 2002; Hikosaka et al., 1996; Jacobacci et al., 2020; Penhune & Steele, 2012). In contrast, the recruitment of the right M1 in the schema-incompatible group might reflect increased use of control and attentional processes potentially induced by the ordinal incongruency. The right hemisphere in general is known to be critical for the control of visuospatial processing and spatial attention (Corballis, 2003; Coull & Nobre, 1998; Winstein & Pohl, 1995), and the primary motor cortex has also been shown to participate in attention to movement and motor imagery (Binkofski et al., 2002; Johansen-Berg & Matthews, 2002; Lotze et al., 1999; Porro et al., 1996; Tagaris et al., 1998; Tomasino et al., 2005, see Bhattacharjee et al., 2021 for a review). Together with the evidence reviewed above that the right M1 is less prone than the left M1 to support learning, greater right M1 activation during learning of a schema-incompatible sequence may reflect a need for increased attention to the visual stimuli when presented in an ordinal framework that is incompatible with previous experience. It should be noted that previous studies reported differences in motor cortical recruitment during motor control in left- versus right-handers, with left-handers displaying less pronounced asymmetry (Kim et al., 1993; Solodkin et al., 2001). We can, therefore, not exclude the possibility that a different pattern of results may be observed in left-handed individuals.

Differences in activation between the compatible and incompatible groups were also observed in the cerebellum and in the hippocampus. Interestingly, these effects were driven by negative parameter estimates in the schema-incompatible group while estimates were around zero in the schema-compatible group. These results suggest that the cerebellum and the hippocampus were more *deactivated* during task practice in the incompatible than in the compatible group. Our results, therefore, support the hypothesis that hippocampal responses are modulated by schema compatibility in the motor memory domain but with a direction that was opposite to our expectations. Indeed, in the declarative memory domain, hippocampal involvement has been described to be *greater* during schema-incompatible than during schema-compatible learning (van Kesteren et al., 2009, 2013). Additionally, recent neuroimaging work suggests that the hippocampus plays a critical role in the acquisition of new motor sequences (Albouy et al., 2013, 2015; Fernández-Seara et al., 2009; Jacobacci et al., 2020) and represents ordinal information about movements in a learned sequence (Dolfen et al., 2024). According to these previous studies, we hypothesized that the hippocampus would be involved in the learning of the schema-incompatible motor sequence but not in the learning of the schema-compatible sequence which was expected to be rapidly assimilated into cortical storage. We, therefore, predicted that the practice of the incompatible sequence would result in *increased* task-related hippocampal activity. In contrast to these expectations, the current results point to greater *deactivation* of the hippocampus in the schema-incompatible group. An alternative explanation for the observed results is that hippocampal activity was *increased* at *rest* as compared with *practice* in the schema-incompatible, compared with schema-compatible, group (see negative beta values in Fig. 4). Recently, increased attention has been paid to periods of quiet rest interleaved with task practice during sequence learning as they are proposed to host fast offline motor memory consolidation (Bönstrup et al., 2019). Interestingly, these rest periods have been associated with hippocampal responses that are thought to reflect the reactivation of motor task-related patterns (Buch et al., 2021; Gann et al., 2023 Jacobacci et al., 2020). Based on this previous evidence, it is, therefore, tempting to speculate that the pattern of hippocampal results reflects increased hippocampal reactivations of the new—incompatible—motor sequence task during rest. This is, however, speculative and warrants further investigation into the reactivation of brain patterns elicited by schema-incompatible (vs. schema-compatible) motor learning.

Our connectivity analyses consistently showed that both the left and right M1 were less connected to other task-relevant brain regions during sequence practice (as compared with rest) in a schema-compatible, as compared with schema-incompatible, context. Specifically, the connectivity between the left M1 and a set of brain regions including motor cortical, striatal, hippocampal, and cerebellar areas was decreased under schema-compatible conditions. An analogous pattern of reduced task-related connectivity was also observed in the compatible group between the right M1 and the right cerebellum. The connectivity results observed in the current study are generally in line with previous observations of decreased connectivity in schema-congruent conditions in the declarative memory domain. Specifically, previous research has reported decreased hippocampo-cortical (prefrontal) connectivity during the encoding of schema-compatible, compared with schema-incompatible, novel movie scenes (van Kesteren et al., 2010) and novel factual information (van Kesteren et al., 2014). These results are thought to reflect a decreased need for crosstalk between distant brain regions (in particular between the hippocampus and the prefrontal cortex) in schema-compatible conditions, whereby integration of novel information into memory occurs directly in the neocortex, effectively bypassing hippocampal involvement (van Kesteren et al., 2010; Sharon et al., 2011). The current results extend these findings to the motor memory domain and suggest that schema compatibility resulted in a decreased crosstalk between the motor cortex and distant task-relevant brain regions including the hippocampus, striatum, and cerebellum. In the motor domain, such connectivity decreases are usually observed as learning progresses (Tzvi et al., 2014, 2015), while initial motor learning is generally associated with increased inter- and intra-hemispheric functional coupling between the sensorimotor, premotor, and supplementary motor cortices (Sun et al., 2007) and between the hippocampus and the striatum (Albouy et al., 2013). These results suggest that schema compatibility might have accelerated network segregation processes that are usually observed as a function of practice (Tzvi et al., 2014, 2015). As above, an alternative explanation for the observed results is that connectivity was *increased* at *rest* as compared with *practice* in the schema-compatible, compared with schema-incompatible, group (see beta estimates in Fig. 5). This alternative explanation is in line with previous studies reporting increased functional connectivity *at rest* in hippocampo-frontal networks after encoding of schema-compatible material (Liu et al., 2018; Schlichting & Preston, 2016; but see van Kesteren et al., 2010 for a different account). Such connectivity increase has been associated with greater coarseness of schema-compatible memories (Audrain & McAndrews, 2022), with the loss of detail suggesting increased consolidation and reliance on neocortical retrieval for schema-compatible information (Sekeres et al., 2018).

The results of our key-level multivariate pattern analyses revealed that, in the bilateral primary motor and premotor cortices, pattern similarity was significantly increased for those sequence elements neighbouring the 1^st^ novel key in the sequence, but only when practiced in a schema-compatible, compared with schema-incompatible context. The heightened stability of response to the 1^st^ novel key in the sequence (i.e., to the first element not belonging to the previously acquired schema), together with the increased left M1 recruitment observed during overall sequence practice in the compatible group, suggests a role for motor cortices in the rapid integration of novel schema-compatible information into motor memory. It should be noted that our current design does not allow us to establish the nature of the information represented by the multivariate patterns. The observed increased consistency of motor cortical responses to the 1^st^ novel key may thus reflect these regions’ consistent response to the key itself, its ordinal position in the sequence, or the binding of the two resulting in the sequence element being perceived as novel in the context of the previously acquired cognitive–motor schema. However, previous studies using multivariate pattern analyses demonstrated that multivoxel patterns in M1 and the premotor cortex represent information about both individual finger movements (Berlot et al., 2020; Dolfen et al., 2024; Yokoi & Diedrichsen, 2019; Yokoi et al., 2018) and movement chunks and entire sequences (Kornysheva & Diedrichsen, 2014; Yokoi & Diedrichsen, 2019). Additionally, the premotor cortex is known to carry ordinal position information in streams of movements (Dolfen et al., 2024; Kornysheva & Diedrichsen, 2014) which may provide a temporal scaffold in which movements are integrated during task practice. We could thus speculate that M1 and the premotor cortex may play a role in the adaptation of the previously acquired sequence schema to accommodate new movements into memory if the new material to learn is compatible with previous experience. We propose that in the current study, the specific responses of M1 and the premotor cortex around the 1^st^ novel key in the schema-compatible sequence may reflect these regions’ capacity to adapt the previously learned ordinal-based cognitive–motor schema (King et al., 2019) to assimilate the novel material. It is unclear why such heightened similarity was not also observed for the 2^nd^ novel element in the sequence. It could be argued that the integration process was initiated upon exposure to the first novel element in the sequence stream, and that the multivoxel activation patterns of subsequent novel elements were less consistent. It is, however, important to note that, given the strong main effect of ordinal position observed across all ROIs in this dataset, it is difficult to compare patterns related to the 2^nd^ novel key between the two experimental groups as these keys are presented in different ordinal positions (including one edge position). Therefore, we cannot exclude the possibility that ordinal coding may have masked any differences elicited by schema compatibility on the 2^nd^ novel key.

The multivariate MRI analyses also show that when practice takes place in a schema-incompatible context, the left (but not the right) M1 and the premotor cortex displayed higher similarity for the 1^st^ element of the sequence. A first key effect was previously reported in M1, with M1 activation patterns largely depending on the sequence’s starting element (Yokoi et al., 2018). The current results extend these findings and show that this first key effect is (i) lateralized to the left M1, (ii) also observed in the premotor cortex, and (iii) modulated by previous experience as it is more pronounced when motor sequences are practiced in a context that is incompatible (as compared with compatible) with a previously acquired cognitive–motor schema. Consistent with the motor and premotor cortices’ sensitivity to novel keys in a compatible context discussed above, and considering that the schema-incompatible sequence, contrary to the schema-compatible sequence, is different from the previously learned schema from the very 1^st^ keypress, the first key effect observed in our study may thus reflect an ordinal/key mapping mismatch. However, as no increase in similarity was observed in the schema-incompatible context after the first key, even though all other keys presented a similar ordinal mismatch, we speculate that this effect more likely reflects the salience of the ordinal mismatch at the start of the sequence. Indeed, in the incompatible group, no increases of pattern similarity were observed during or following performance of the novel keys. It could be argued that any increased salience of the incompatible sequence may have been limited to the pre-test performed outside of the scanner, as performance in the MRI runs did not differ between groups. However, the lack of behavioural difference, as discussed above, is likely related to the slower task pace in-scanner, and neural effects of salience could have outlasted its behavioural manifestation. We argue that both the first key salience effect observed on the multivoxel patterns of the left M1 and the premotor cortex as well as the more general incompatibility effect observed on the amplitude of the BOLD signal in the right M1 may reflect increased attentional need during sequence performance in a schema-incompatible context.

As discussed above, one limitation of the current study is the fixed relationship between each key pressed and its ordinal position in the sequence, which made it impossible to disentangle individual contributions of key and ordinal information to the observed pattern of results. We attempted to mitigate this limitation with control analyses examining the influence of key information on our data (see Supplementary Material); however, these analyses were conducted on a separate dataset using a within-subject design (Dolfen et al., 2024) and thus could not entirely account for key effects in the current study that used a between-subject design. These control analyses—as well as the multivariate results presented in the manuscript—should, therefore, be interpreted with caution and future studies should include random sequence conditions, as in the work of Dolfen et al. (2024), to address this limitation. Additionally, future research could consider alternative motor-schema conceptualization. For example, schema manipulations across memory domains could be investigated via the use of a common sequential structure applied to both declarative and motor sequences. Recent studies indeed suggest that sequential structures can be abstracted and applied to both object (words, images) and action sequences, with the hippocampus carrying information about items in their learned ordinal position, independent of their nature (Mutanen et al., 2020; Temudo et al., 2025). A manipulation of this abstract, cross-domain schema may be more suitable to induce a modulation of hippocampal patterns. Another limitation of the current work is related to task pace in the MRI scanner as the investigation of multivariate patterns at the key level required us to slow down task pace during the MRI runs. As brain activity patterns were demonstrated to be more distinct when sequences were performed at full speed rather than in a paced manner (Berlot et al., 2020), future studies may include both full-speed runs, optimized for sequence-level analyses, and paced runs, optimized for key-level analyses.

In summary, our results indicate that schema-mediated motor learning is supported by increased activity in the left primary motor cortex and a decreased crosstalk between the motor cortex and distant task-relevant brain regions including the hippocampus, striatum, and cerebellum. Our findings also suggest that multivoxel activation patterns can be altered in the primary and the premotor cortices to assimilate new movements that are compatible with the previously acquired cognitive–motor schema. These results deepen our understanding of the neural processes underlying schema-mediated learning in the motor memory domain and suggest the involvement of motor cortical regions in schema-mediated integration of novel movements into memory.

## Supplementary Material

Supplementary Material

## Data Availability

Raw data, analyzed data corresponding to the figures presented in the text, and the scripts used to produce them are publicly available on OSF (https://osf.io/xnrg8).
