# Supplementary material for: Motor cortical areas facilitate schema-mediated integration of new motor information into memory
Source: Imaging Neurosci (Camb). 2026 Apr 13;4:IMAG.a.1203. doi: 10.1162/IMAG.a.1203 (PMC13081739; doi:10.1162/IMAG.a.1203)
Supplement: Supplementary Material [file IMAG.a.1203_supp.pdf]

Supplementary material

Participant characteristics

Participant characteristics, vigilance, sleep duration and quality for the nights preceding the experimental sessions are reported in Table S1 and did not differ between experimental groups.

Table S1: Participant demographics, sleep and vigilance data for each experimental group.

	Group mean (SD)				
**Variable**	**COMP**	**INCOMP**	**t**	**p**	**Cohen's d**
N	30	30			
Female (n)	20	20			
Age (years)	23.1 (2.8)	23.5 (2.7)	-0.52	0.61	-0.134
BAI score	3.0 (3.1)	3.2 (3.3)	-0.24	0.81	-0.063
BDI score	4.3 (3.8)	3.6 (3.9)	0.70	0.49	0.181
Handedness score	80.8 (14.6)	84.0 (16.0)	-0.80	0.43	-0.207
PSQI score	3.3 (1.8)	2.9 (1.3)	1.16	0.25	0.298
Chronotype score	50.6 (7.0)	50.9 (7.0)	-0.20	0.84	-0.052
Average sleep duration, 3 nights prior S1 (hours)	8:23 (0:44)	8:22 (0:46)	0.10	0.92	0.025
Sleep duration, night prior S2 (hours)	8:38 (0:58)	8:38 (0:52)	0.00	>0.99	0.000
Session 1 SMS duration (hours)	8:09 (1:04)	8:13 (0:41)	-0.27	0.79	-0.069
Session 2 SMS duration (hours)	8:22 (0:56)	8:11 (0:48)	0.82	0.42	0.212
Session 1 SMS quality	4.0 (0.6)	4.0 (0.7)	0.10	0.92	0.025
Session 2 SMS quality	4.0 (0.5)	4.1 (0.6)	-0.68	0.50	-0.175
Session 1 PVT (ms)	298 (21)	287 (30)	1.73	0.09	0.446
Session 2 PVT (ms)	302 (25)	290 (30)	1.81	0.08	0.467

Group means are presented, with standard deviation in parentheses. Average sleep duration for the 3 nights prior to S1 was assessed via self-reported sleep diaries. Sleep duration for the night prior to S2 was assessed via visual inspection of actigraphy data extracted from wrist-mounted device. Statistical differences between groups were assessed with independent samples t-tests. BAI = Beck Anxiety Inventory (Beck et al., 1988); BDI = Beck Depression Inventory (Beck et al., 1996); PSQI = Pittsburgh Sleep Quality Index (Buysse et al., 1989); SMS = St. Mary's Hospital Sleep Questionnaire (Ellis et al., 1981). Degrees of freedom=56 for Average sleep duration, 3 nights prior to S1, and for Sleep duration, night prior to S2; degrees of freedom=58 for all other variables. Cutoff scores for inclusion were: BAI<16; BDI<19; Handedness>40; PSQI<8; Chronotype 30<n<70.

Random SRTT performance

Table S2: Output of the statistical analyses assessing group differences in performance on the pseudo-random SRT task administered prior to the sequential SRT task in Session 1.

Effect	df	F	p	Partial η^2
**A. Response Time**				
Block	2.83,163.99	10.08	**<0.001***	0.148
Block x Group	2.83,163.99	0.48	0.69	0.008
Group	1,58	0.27	0.61	0.005
**B. Accuracy**				
Block	3,174	1.11	0.35	0.019
Block x Group	3,174	0.18	0.91	0.003
Group	1,58	0.06	0.82	0.001

Separate 4 (Block) by 2 (Group) ANOVAs were run per each variable (A: Response Time; B: Accuracy). The significant effect of block on RT reflects increased task familiarization with practice. The lack of group effect or block x group interaction indicate that general motor execution did not differ between experimental groups at baseline. Df=degrees of freedom. Significant p-values are marked with an asterisk and bold font.

Small volume correction for univariate MRI analyses

Table S3: Coordinates used for small volume correction for the data presented in Table 3 of the main text.

Area	x	y	z	Reference coordinate
Superior frontal cortex	±22	12	54	(Penhune & Doyon, 2002)
Primary sensorimotor cortex	±36.2±3.0	-22.3 ±4.6	57.0±6.1	(Lehéricy et al., 2006)
Supplementary motor area	±10	-22	58	(Penhune & Doyon, 2005)
Precuneus	±20	-52	50	(Fischer et al., 2005)
Intra parietal sulcus	±48	-50	60	(Albouy et al., 2008)
Cerebellar lobules IV-V	±12	-58	-10	(Destrebecqz et al., 2005)
	-18	-44	-18	(Fischer et al., 2005)
Cerebellar lobule VI	28	-54	-28	(Dolfen et al., 2021)
Cerebellar lobule VIII	±22	-62	-48	(Fischer et al., 2005)
Putamen	28	-14	-8	(Albouy et al., 2008)
Hippocampus	-18	-16	-14	(Strange et al., 1999)
	±32	-42	-4	(Albouy et al., 2008)
	±42	-34	-12	(Albouy et al., 2008)

ROI size for multivariate MRI analyses

Table S4: Average number of voxels per each ROI used in representational similarity analyses per experimental group.

	Group mean (SD)				
**ROI**	**COMP**	**INCOMP**	**t**	**p**	**Cohen's d**
Hippocampus	877 (103)	874 (86)	0.12	0.91	0.031
Putamen	999 (135)	1007 (112)	-0.27	0.79	-0.070
Left M1	782 (89)	792 (91)	-0.42	0.68	-0.110
Right M1	671 (80)	689 (74)	-0.89	0.38	-0.232
Premotor cortex	1278 (155)	1285 (154)	-0.18	0.86	-0.046
aSPL	710 (105)	727 (86)	-0.68	0.50	-0.177
Caudate nucleus	839 (118)	823 (69)	0.64	0.53	0.165
Angular gyrus	3842 (502)	3822 (361)	0.18	0.86	0.047
mPFC	1585 (197)	1563 (183)	0.46	0.65	0.119

Group means are presented, with standard deviation in parentheses. Statistical differences between groups were assessed with independent samples t-tests. Degrees of freedom=57 for all tests. The angular gyrus and mPFC ROIs were considered exploratory analyses.

Neuroimaging results: univariate analyses

Task practice > Rest contrast across both experimental groups

We examined which brain regions were recruited during task practice, compared to rest, across both experimental groups. As expected, task practice recruited a wide bilateral network of subcortical (putamen), cortical (M1, supplementary motor area (SMA), superior parietal lobule) and cerebellar regions across the two experimental groups which is in line with previous literature (Dayan & Cohen, 2011; Doyon et al., 2003). The corresponding results are reported in Table S5.

Table S5: Functional imaging results for Session 2 SRTT practice versus rest, across both experimental groups.

Area	x	y	z	k	T
Supplementary motor area	-6	0	56	30263	15.76
R Primary motor cortex	36	-16	60		14.95
L Primary motor cortex	-40	-38	48		18.18
L Superior parietal lobule	-32	-52	54		14.57
R Superior parietal lobule	34	-48	56	7306	11.47
R Cerebellum superior	28	-62	-26	10442	14.28
R Cerebellum posterior	28	-68	-52		11.81
L Cerebellum superior	-18	-54	-24		14.11
L Cerebellum posterior	-20	-64	-48		13.35
L Putamen	-24	2	4	5102	12.46
R Putamen	26	2	4		11.56

Significance level set at p<0.05 corrected on the whole brain using family-wise error correction (FWE). K represents cluster size (# of voxels).

Comparisons between experimental groups, activations outside regions of interest

Table S6: Functional imaging results for Session 2 SRTT practice: between-group comparisons (a,b) and PPI analyses (c,d) based on seed regions identified in (a,b).

Area	x	y	z	k	T
**a. Main effect of schema compatibility (COMP > INCOMP)**					
R Calcarine sulcus	20	-72	6	1366	4.07
L Cuneus	-8	-92	26	25	3.72
L Medial occipital cortex	-28	-76	-2	20	3.45
L Lingual gyrus	-14	-50	0	31	3.41
**b. Main effect of schema incompatibility (INCOMP > COMP)**					
L Anterior frontal cortex	-44	48	16	27	3.51
**c. Psycho-physiological interaction, seed region left primary motor cortex (INCOMP > COMP)**					
R Frontal eye fields	2	34	50	179	4.32
R Medial temporal gyrus	52	-34	-16	29	3.64
R Superior frontal cortex	30	66	8	76	3.73
L Superior frontal cortex	-26	68	2	27	3.66
L Fusiform gyrus	-64	-54	-4	31	3.54
L Anterior prefrontal cortex	-40	56	-8	8	3.44
R Medial temporal gyrus	54	-6	-22	7	3.36
L Medial temporal gyrus	-62	-20	-12	9	3.34
**d. Psycho-physiological interaction, seed region right primary motor cortex (INCOMP > COMP)**					
R Occipital cortex	24	-92	8	7	3.36

Only activations outside regions of interest are reported here, refer to Table 3 in the main text for activations within regions of interest. Significance level set at p<0.001 uncorrected.

Neuroimaging results: multivariate pattern analyses

Influence of key information on pattern similarity

In this study, both schema-compatible and -incompatible experimental groups practiced the same sequence of movements but with a different starting point. Key information was therefore matched across groups (i.e., the same series of keys were pressed in both groups) but the ordinal position of the keys in the sequence stream differed between groups (e.g., key 4 was pressed in ordinal position 1 in COMP and 6 in INCOMP). As our previous work indicates a pronounced effect of ordinal position on similarity values (Dolfen et al., 2024), we accounted for this potential effect by matching ordinal positions when comparing the data between groups in the current study (see *group* x *ordinal position* ANOVAs reported in the main text). However, this does not allow us to control for the potential effect of key information on similarity values.

To investigate how multivariate patterns may vary as a function of key information, we extracted from our previously published data (Dolfen et al., 2024) pattern similarity values in a random SRT task where key and ordinal information are not associated across trials. This allowed us to examine the specific effect of key information (irrespective of ordinal information) on the amplitude of similarity patterns in the specific ROIs used in the current study (see "Abbreviated methods" below, and see Dolfen et al., 2024 for details). The results of these analyses are reported in Table S7. The repeated measures ANOVA

using *key* as a within-subject factor did not show any main effect of key on the PMC, hippocampus, putamen and aSPL, but there was a significant key effect in the left and right M1. Table S8 presents all the follow-up pairwise comparisons for these ROIs. This suggests that, while the pattern of results presented in the original manuscript for PMC, aSPL, putamen and hippocampus are unlikely to be explained by key effects, patterns observed in M1 might have been influenced by key information. To better address this possibility, we re-examined the results presented in the main text on the left and right M1 considering the key effects reported above.

Note that, for completeness, we also report in Table S7 the results of the repeated measures ANOVA on the random SRTT dataset using *ordinal position* as a within-subject factor. As expected, these results show a significant ordinal position effect on all the ROIs examined in the current study. Similar to data collected in the current study, this effect was generally due to increased similarity on edge positions as the average similarity of positions I and VIII was greater than that of positions II-VII in all ROIs (paired-samples t-tests, all ROIs $p<0.01$).

Table S7: Results of multivariate pattern analyses for the main effect of Ordinal position (Ord; I through VIII) or Key (1 through 8) on pattern similarity extracted from random SRT data (Dolfen et al., 2024), per each ROI examined in the current manuscript.

ROI	Main effect Ord				Main effect Key			
	**df**	**F**	p_{corr}	**Part η^2**	**df**	**F**	p_{corr}	**Part η^2**
**L M1**	3.06,97.77	36.10	**<0.001***	0.530	5.07,162.25	6.79	**<0.003***	0.175
**R M1**	4.46,142.78	40.79	**<0.001***	0.560	7,224	4.87	**<0.003***	0.132
**PMC**	3.57,114.15	62.15	**<0.001***	0.660	7,224	1.17	0.48	0.035
**aSPL**	3.57,114.19	13.93	**<0.001***	0.303	7,224	0.85	0.55	0.026
**HC**	3.71,118.56	2.84	**0.03***	0.082	7,224	1.01	0.51	0.031
**Put**	4.59,146.71	6.46	**<0.001***	0.168	7,224	2.02	0.11	0.059

L/R M1 = left/right primary motor cortex; PMC = premotor cortex; aSPL = anterior superior parietal lobule; HC = hippocampus; Put = putamen; df = degrees of freedom; part η^2 = partial eta squared. P_{corr} indicates the p-value corrected for multiple comparisons (FDR correction for the number of ROIs). Significant p-values are marked with an asterisk and bold font.

Table S8: Results of paired-samples t-tests following up on the key effect reported above and contrasting pattern similarity for each of the 8 keys extracted from random SRT data (Dolfen et al., 2024), for the left and right M1 ROIs. Comparisons and direction of difference are indicated in the column Keys; significant differences are indicated with an asterisk.

Keys	Left M1			Keys	Right M1		
	**t**	p_{corr}	**Cohen's d**		**t**	p_{corr}	**Cohen's d**
**1>2***	3.32	**0.01***	0.579	**1>2***	2.58	**0.046***	0.450
1=3	-2.03	0.08	-0.352	**1>3***	2.79	**0.03***	0.486
1=4	2.37	0.06	0.412	**1>4***	3.41	**0.02***	0.594
**1>5***	2.53	**0.04***	0.440	1=5	-0.94	0.45	-0.164
**1>6***	4.60	**<0.005***	0.800	1=6	-0.82	0.47	-0.143
1=7	-0.59	0.67	-0.103	1=7	1.15	0.40	0.200
1=8	2.24	0.06	0.389	1=8	0.14	0.92	0.025
**2<3***	-5.57	**<0.005***	-0.969	2=3	0.07	0.95	0.012
2=4	-0.60	0.67	-0.105	2=4	0.84	0.47	0.147
2=5	-0.17	0.87	-0.030	**2<5***	-2.84	**0.03***	-0.494
2=6	1.56	0.18	0.271	**2<6***	-3.19	**0.02***	-0.555
**2<7***	-3.09	**0.01***	-0.537	2=7	-1.26	0.36	-0.219
2=8	-0.91	0.49	-0.159	2=8	-2.01	0.11	-0.349
**3>4***	3.12	**0.01***	0.543	3=4	0.87	0.47	0.151
**3>5***	3.65	**<0.005***	0.636	**3<5***	-2.49	**0.046***	-0.433
**3>6***	5.91	**<0.005***	1.029	**3<6***	-3.07	**0.02***	-0.534

3=7	0.46	0.70	0.079	3=7	-1.36	0.32	-0.237
**3>8***	3.52	**0.005***	0.613	3=8	-1.88	0.13	-0.327
4=5	0.53	0.67	0.092	**4<5***	-4.37	**<0.01***	-0.760
4=6	2.33	0.06	0.405	**4<6***	-4.14	**<0.01***	-0.721
4=7	-2.13	0.07	-0.371	**4<7***	-2.51	**0.046***	-0.436
4=8	-0.16	0.87	-0.028	**4<8***	-3.12	**0.02***	-0.543
5=6	1.79	0.12	0.312	5=6	0.22	0.89	0.039
**5<7***	-2.53	**0.04***	-0.440	5=7	2.29	0.07	0.399
5=8	-0.54	0.67	-0.094	5=8	1.00	0.45	0.174
**6<7***	-3.88	**<0.005***	-0.676	6=7	1.94	0.12	0.338
6=8	-2.30	0.06	-0.400	6=8	1.01	0.45	0.177
7=8	2.12	0.07	0.370	7=8	-0.98	0.45	-0.170

L/R M1 = left/right primary motor cortex. Degrees of freedom = 32 for all comparisons. P_{corr} indicates the p-value corrected for multiple comparisons (FDR correction for the number of comparisons in each ROI). Significant p-values are marked with an asterisk and bold font.

We then revisit the between-group differences reported in our dataset on the left and right M1 considering how different key information might have influenced the results. Our first observation was a greater similarity in patterns in the INCOMP as compared to the COMP group in the left M1 for the first key presented in the sequence stream (key-pair 4-8). As these specific keys did not elicit different patterns in the random SRTT data (see Table S8), we suggest that the observed group differences in the current dataset are likely driven by the experimental manipulation (INCOMP vs. COMP).

The second observation we report in the main text for both the left and right M1 is an increase in similarity in the COMP group, as compared to the INCOMP group, around the introduction of the 1st novel key in the sequence (i.e., 2nd, 3rd and 4th sequence elements, which corresponds to the key pairs 7-6, 2-3, 8-5). Table S8 above suggests that key information in the left M1 is unlikely to have influenced the results observed for pairs 2-3 (similarity difference is in the opposite direction in random data) and 8-5 (no difference in random data) but might have inflated the results observed for pair 7-6 (similar differences observed in random data). Table S8 also indicates that key information is unlikely to have influenced these results in the right M1 as none of the pairs listed above showed different similarity values in random data.

It should be noted that the dataset used for these control analyses (Dolfen et al., 2024) is based on within-subject pattern comparisons, rather than between-subject comparisons as in the current study. Given the difference in the examined variable, these analyses should therefore be interpreted with caution, and cannot completely exclude an influence of key information on our current data. However, analyses of the random SRTT data of our previous independent sample overall suggest that key information may have influenced only a limited subset of the group differences reported in the main text (i.e., differences observed in ordinal position II in the left M1).

Abbreviated methods

The random SRT data reported above were collected in a previous study from our group (see Dolfen et al., 2024, for details). In brief, the participants included in the study were healthy young adults (N=33; 20 female; mean age 23.4 years). The inclusion criteria were identical to those of the current study, with the exception that we excluded extreme morning or evening chronotypes in the current study (Horne & Ostberg, 1976). Participants were invited to the laboratory for a single experimental session, during which they performed a SRT task similar to that employed in the current experiment. Participants first learned a fixed 8-element sequence of finger movements outside the MRI scanner (20 practice blocks of 48 keypresses each; identical sequence to that performed in Session 1 of our current experiment) before performing both the learned sequence and random sequences of movements in an interleaved fashion while fMRI was collected (8 runs of practice, each consisting of 48 keypresses each of random and fixed sequences). The analyses reported above focus on the random SRTT condition, where there was no repeating sequence but each key was pressed once every eight keypresses in a random order; consequently, key and ordinal position information did not have a fixed

association. fMRI data was acquired and pre-processed with identical parameters to those of the current study. fMRI analyses were identical to those of the current study, with the exception that similarity patterns were calculated over multiple presentations of random rather than fixed sequences, by correlating t-values extracted from the same key (presented in different ordinal positions), or the same ordinal position (corresponding to different pressed keys), across task runs.

Analyses on exploratory ROIs

We report here MVPA analyses (*group* x *ordinal position* ANOVAs), analogous to those presented in the manuscript, for three exploratory ROIs: the caudate nucleus, the angular gyrus, and the medial prefrontal cortex (mPFC).

Despite its role in motor learning, previous work examining multivariate patterns elicited by performance of both learned sequences and random movements revealed that the **caudate nucleus** did not show preferential coding for key information, ordinal position information, or their association in a learned sequence (Dolfen et al., 2024). Consequently, we did not expect caudate nucleus multivoxel patterns in this study to be influenced by schema-compatibility of the sequence learning context. Results (Table S9, Figure S1) reveal a significant main effect of ordinal position but no group x ordinal position interaction. These results are in line with our prior work and do not suggest that schema-compatibility significantly affected multivariate patterns in the caudate nucleus.

Although this study is to our knowledge the first investigation of schema-mediated learning facilitation in the motor memory domain, this effect is well studied in the declarative domain, where it was shown to critically involve the **angular gyrus** (Wagner et al., 2015) and the **medial prefrontal cortex (mPFC)** (van Kesteren et al., 2013). Recently, increasing evidence has suggested that the declarative and motor memory systems share common processes and neural substrates; indeed, the hippocampus was shown to be involved in motor sequence learning (Albouy et al., 2008, 2013; Fernández-Seara et al., 2009) while striatal activation was reported in hippocampal-dependent spatial and associative memory tasks (Brodt, Schönauer, et al., 2023; Doeller et al., 2008). Consequently, we conducted exploratory multivoxel pattern similarity analyses of these two ROIs traditionally associated with the schema memory model in the declarative domain.

The results of the *group* x *ordinal position* ANOVAs are reported in Table S9 and corresponding Figure S1. In both ROIs, there was a significant main effect of ordinal position, but no main effect of group or interaction of group and ordinal position. These results suggest that these regions are not sensitive to the schema-compatibility of the motor learning context. Thus, although both the mPFC and angular gyrus were shown to play a key role in schema-mediated learning in the declarative domain (Bonnici et al., 2012; Frankland & Bontempi, 2005; Takashima et al., 2006, 2007; Wagner et al., 2015), our results suggest that they are not involved in the corresponding process in the motor domain.

Table S9: Results of exploratory multivariate pattern analyses in three exploratory ROIs (caudate nucleus, angular gyrus, medial prefrontal cortex) for the 2 groups (COMP/INCOMP) x 8 ordinal positions (I through VIII), and the 2 groups x 8 keys (1 through 8) ANOVAs.

ROI	Main effect Group				Main effect Ord				Group x Ord interaction			
	df	F	p	Part η^2	df	F	p	Part η^2	df	F	p	Part η^2
Caud	1,57	0.01	0.93	0.000	2.99,170.33	13.40	**<0.001***	0.190	2.99,170.33	1.15	0.33	0.020
Ang	1,57	0.25	0.62	0.004	4.42,252.19	16.76	**<0.001***	0.227	4.42,252.19	1.06	0.38	0.018
mPFC	1,57	0.00	0.97	0.000	4.02,229.06	3.11	**0.02***	0.052	4.02,229.06	2.11	0.08	0.036

Caud = caudate nucleus; Ang = angular gyrus; mPFC = medial prefrontal cortex; df = degrees of freedom; part η2 = partial eta squared. Significant p-values are marked with an asterisk and bold font.

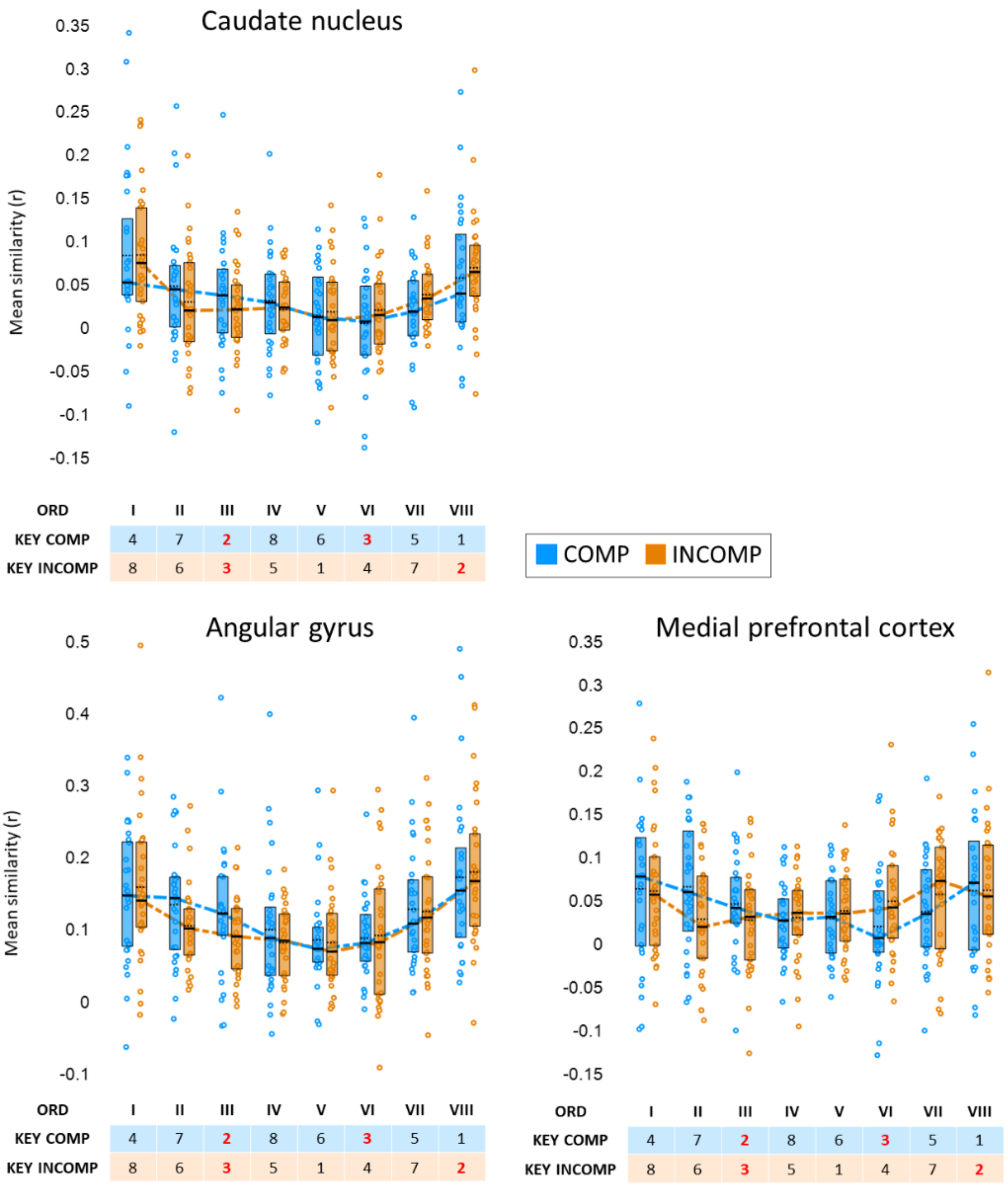

Figure S1: Mean pattern similarity per key/ordinal position pairing in three exploratory ROIs (caudate nucleus, angular gyrus, medial prefrontal cortex). Black horizontal bars (full) indicate the median and black horizontal bars (dotted) the mean. Boxes represent the interquartile range (IQR). Coloured dashed lines connect the medians in each experimental group. Coloured circles represent individual data.

Supplementary material – additional references

Brodt, S., Schönauer, M., Seewald, A., Beck, J., Erb, M., Scheffler, K., & Gais, S. (2023). Memory systems integration in sleep complements rapid systems consolidation in wakefulness. Bio-Rxiv, 2023.03.09.531360. https://doi.org/10.1101/2023.03.09.531360

Destrebecqz, A., Peigneux, P., Laureys, S., Degueldre, C., Fiore, G. Del, Aerts, J., Luxen, A., Van Der Linden, M., Cleeremans, A., & Maquet, P. (2005). The neural correlates of implicit and explicit sequence learning: Interacting networks revealed by the process dissociation procedure. Learning & Memory, 12(5), 480–490. https://doi.org/10.1101/LM.95605

Doeller, C. F., King, J. A., & Burgess, N. (2008). Parallel striatal and hippocampal systems for land-marks and boundaries in spatial memory. Proceedings of the National Academy of Sciences of the United States of America, 105(15), 5915–5920. https://doi.org/10.1073/PNAS.0801489105

Fernández-Seara, M. A., Aznárez-Sanado, M., Mengual, E., Loayza, F. R., & Pastor, M. A. (2009). Continuous performance of a novel motor sequence leads to highly correlated striatal and hippocampal perfusion increases. NeuroImage, 47(4), 1797–1808. https://doi.org/10.1016/J.NEUROIMAGE.2009.05.061

Lehéricy, S., Bardinet, E., Tremblay, L., Van De Moortele, P. F., Pochon, J. B., Dormont, D., Kim, D. S., Yelnik, J., & Ugurbil, K. (2006). Motor control in basal ganglia circuits using fMRI and brain atlas approaches. Cerebral Cortex, 16(2), 149–161. https://doi.org/10.1093/CERCOR/BHI089

Penhune, V. B., & Doyon, J. (2002). Dynamic Cortical and Subcortical Networks in Learning and Delayed Recall of Timed Motor Sequences. Journal of Neuroscience, 22(4), 1397–1406. https://doi.org/10.1523/JNEUROSCI.22-04-01397.2002

Penhune, V. B., & Doyon, J. (2005). Cerebellum and M1 interaction during early learning of timed motor sequences. NeuroImage, 26(3), 801–812. https://doi.org/10.1016/J.NEUROIMAGE.2005.02.041

Strange, B. A., Fletcher, P. C., Henson, R. N. A., Friston, K. J., & Dolan, R. J. (1999). Segregating the functions of human hippocampus. Proceedings of the National Academy of Sciences, 96(7), 4034–4039. https://doi.org/10.1073/PNAS.96.7.4034
